# SNRPB/CCNB1 axis promotes hepatocellular carcinoma progression and cisplatin resistance through enhancing lipid metabolism reprogramming

**DOI:** 10.1186/s13046-025-03463-y

**Published:** 2025-07-18

**Authors:** Xin Jin, Xigan He, Runze Huang, Qinyu Liu, Lei Wang, Xuanci Bai, Yibin Wu, Yixiu Wang, Ziting Jiang, Yi Shi, Gautam Sethi, Lu Wang, Weiping Zhu

**Affiliations:** 1 Department of Hepatic Surgery, Fudan University Shanghai Cancer Center, Shanghai Medical College, Fudan University, Shanghai, 200032 P.R. China; 2 Department of Oncology, Shanghai Medical College, Fudan University, Shanghai, 200032 PR China; 3 Department of Pathology, Fudan University Shanghai Cancer Center, Shanghai Medical College, Fudan University, Shanghai, 200032 PR China; 4 Department of Endoscopy, Fudan University Shanghai Cancer Center, Shanghai Medical College, Fudan University, Shanghai, 200032 PR China; 5 Bio-X Institutes, Key Laboratory for the Genetics of Developmental and Neuropsychiatric Disorders, Shanghai Jiaotong University, Shanghai, 200030 PR China; 6 Department of Pharmacology, Yong Loo Lin School of Medicine, National University of Singapore, Singapore, 117600 Singapore; 7 NUS Centre for Cancer Research, Yong Loo Lin School of Medicine, National University of Singapore, Singapore, 117699 Singapore

**Keywords:** Hepatocellular carcinoma, Lipid metabolism, SNRPB, Drug resistance

## Abstract

**Background:**

Hepatocellular carcinoma (HCC) is a major cause of cancer-related mortality globally, significantly impacting worldwide health. Hence, identifying key molecular drivers of HCC progression is crucial for enhancing treatment options and prognostic methods. This study explores the function of Small Nuclear Ribonucleoprotein Polypeptides B and B1 (SNRPB) in HCC, unveiling critical pathways that affect the progression of the disease.

**Methods:**

Utilizing multi-dimensional data that integrates bulk RNA sequencing (bulk RNA-seq), single-cell RNA sequencing (scRNA-seq), and spatial transcriptomics (ST) from HCC patients, we have identified SNRPB as a pivotal gene associated with the spliceosome, playing a central role in both tumor initiation and progression. We also investigated the intricate process by which SNRPB influences cyclin B1 (CCNB1) expression through FOXM1-mediated activation, using a combination of bioinformatics, functional assays, Chromatin Immunoprecipitation (ChIP), and Co-Immunoprecipitation (Co-IP) studies. Complementary in vivo experiments and metabolic assays were conducted to explore the relationship between tumor growth and lipid metabolism further. Additionally, evaluations of cisplatin sensitivity were performed, providing an in-depth analysis of influence of SNRPB on HCC.

**Results:**

Across multiple cohorts, SNRPB exhibited a marked upregulation within tumors, correlating significantly with poor prognosis. Knockdown of SNRPB suppressed HCC cell proliferation and migration, while promoting apoptosis. Mechanistically, SNRPB regulated CCNB1 expression *via* FOXM1-mediated transcription, and SNRPB overexpression enhanced lipid metabolism and cisplatin resistance. This increase in drug sensitivity was mediated through alterations in lipid metabolism and the regulatory effects on CCNB1, providing a comprehensive insight into multifaceted role of SNRPB in HCC pathology and potential therapeutic targets. Finally, CCNB1 knockdown reversed the proliferative and tumorigenic effects of SNRPB overexpression in a preclinical HCC model.

**Conclusions:**

SNRPB promoted HCC progression by modulating the FOXM1-CCNB1 axis and lipid metabolism, and could act as a potential therapeutic target to augment chemotherapy sensitivity in HCC.

**Supplementary Information:**

The online version contains supplementary material available at 10.1186/s13046-025-03463-y.

## Introduction

Accounting for approximately 80–90% of liver cancer cases, HCC is the most common type of primary liver cancer [1, 2]. It holds a significant global burden, ranking sixth in incidence and third in cancer-related mortality worldwide [3, 4]. HCC poses a significant public health challenge, particularly in developing countries where hepatitis B virus (HBV) and hepatitis C virus (HCV) infections stands as a pivotal risk element in HCC development [5, 6]. In China alone, nearly half of the global new cases of liver cancer occur each year [7, 8]. Due to the asymptomatic nature of early-stage liver cancer, individuals with HCC frequently are often diagnosed at advanced stages, which limits the applicability of curative surgical resection to a small fraction of the patient population.

While liver transplantation offers a promising curative option, its application is restricted by donor shortages and strict eligibility criteria. Other localized therapies, such as ablation and transarterial chemoembolization (TACE), provide clinical benefits for some patients with advanced HCC but are not curative [9, 10]. Molecularly targeted therapies, including VEGF inhibitors like sorafenib and lenvatinib, have shown efficacy in advanced HCC, yet their therapeutic potential is limited by the development of resistance [11]. Although recent advances in immunotherapy have shown promise, the response rates in HCC remain suboptimal when immunotherapy is used as a monotherapy [12, 13]. Therefore, further investigation into the underlying mechanisms driving the progression of HCC is crucial to identify key molecules and signaling pathways that could improve treatment efficacy and provide additional therapeutic options for HCC patients.

SNRPB is an essential component of the spliceosome complex and is classified within the Sm protein family, which includes other core members such as SNRPD1, SNRPE, and SNRPN [14, 15]. The SNRPB gene is located on chromosome 20 and is expressed across various tissues, playing a key role in the alternative splicing process of pre-mRNA. This process, executed by the spliceosome, involves removing introns and joining exons to generate mature mRNA, which greatly enhances protein diversity and regulates gene expression [16, 17]. SNRPB specifically functions as a critical component of the spliceosome, contributing to the regulation of gene expression through alternative splicing. Recent studies have implicated aberrant SNRPB expression in the pathogenesis of various cancers including lung and cervical cancers, indicating its potential role as a biomarker or therapeutic target in oncology [18, 19]. However, despite these findings, research on the role SNRPB plays in HCC remains limited. Some studies suggested that SNRPB may influence tumor progression in HCC through cell cycle regulatory pathways, although the precise molecular mechanisms involved remined not fully understood [20]. Therefore, characterizing the downstream effects of SNRPB dysregulation in HCC, such as alterations in gene expression profiles or cellular processes, could provide a comprehensive understanding of its impact on tumor biology.

In this study, the process involving bioinformatics analysis, phenotype validation, and detailed mechanistic investigations allowed us to uncover the significant role of SNRPB in HCC progression and its intricate link to lipid metabolic pathways. Firstly, leveraging a multi-omics approach that combines bulk RNA-seq, the scRNA‑seq and ST data, we discerned SNRPB as a key regulator with potential prognostic significance in HCC. Subsequent experiments confirmed that the suppression of SNRPB hindered cell proliferation, migration and colony formation while promoting apoptosis, underscoring the crucial involvement of SNRPB in HCC progression. Additionally, we uncovered that SNRPB enhanced CCNB1 expression through FOXM1-mediated transcriptional activation, contributing to tumor growth. Importantly, SNRPB was also found to modulate lipid metabolism of HCC cells. Finally, we explored the impact of SNRPB on cisplatin sensitivity, revealing that its knockdown enhances cisplatin sensitivity through lipid metabolism and CCNB1 regulation.

## Materials and methods

### scRNA‑seq data analysis

The scRNA-seq data were sourced from two datasets available in the Gene Expression Omnibus (GEO) database (GSE151530^21^ and GSE202642^22^). For quality control (QC), low-quality cells (< 300 genes/cell and > 15% mitochondrial genes) were excluded. Totally 129,401 cells from 43 patients were finally involved for the subsequent analysis. The package Seurat were used to complete basic downstream analysis and visualization. In detail, the data was scored with top 3000 most variable genes across FindVariableFeatures function. Subsequent application of principal component analysis (PCA) facilitated the visualization of cellular diversity within a reduced-dimensional space. To minimize batch effects, we performed the Harmony algorithm to integrate the expression data from disparate samples. The identification and clustering of cell types relied on FindNeighbors and FindCluster function. Finally, the visualization of the cells obtained from the above steps was achieved through uniform manifold approximation and projection (UMAP).

Utilizing the Scillus package, we identified genes that were differentially expressed and performed Gene Set Enrichment Analysis (GSEA) across a spectrum of cell types in both tumorous and normal tissues. The identification of the spliceosome-associated tumor cell cluster was facilitated through the AddModuleScore function in Seurat. This function served as a powerful tool that enabled researchers to assess the activity of specific gene sets within scRNA-seq, illustrating the changes in cellular states and functions. What’s more, CellChat package [23] was used to the inference and visualization of interactions between different cell types. It allowed us to evaluate interaction intensity between ligand-receptor pairs by examining their expression levels across distinct cell types, facilitating the identification of significantly differentially expressed ligand-receptor pairs across specific cell types. In this study, the spliceosome-associated genes were derived from the spliceosome gene set available in the kyoto encyclopedia of genes and genomes (KEGG) database [24].

### Bulk RNA-seq data analysis

The bulk RNA-seq data applied to verify the results in scRNA-seq was retrieved from The Cancer Genome Atlas (TCGA-LIHC) (371 HCC cases and 50 normal controls) and International Cancer Genome Consortium (ICGC-LIRI-JP) (240 HCC cases and 202 normal controls). What’s more, three datasets available in GEO (GSE14520, GSE76247, GSE36376) were also included in the validation set.The clinical information from TCGA was also incorporated to examine the correlations with identified cellular subset, thereby further elucidating its clinical value. In order to validate the results of our analysis, a local cohort from Fudan University Shanghai Cancer Center (FUSCC) was included as an independent validation cohort. The deconvolution algorithm Bisque package [25] was employed to map the identified cluster in scRNA-seq onto bulk RNA-seq profiles to speculate the proportion and functional role of the cluster in HCC.

### ST data analysis

With the ST data sourced from GSE238264 26 and HRA000437 27, we utilized the Seurat package with an integrated mapping approach to project scRNA-seq derived cluster onto ST sections, investigating the spatial characteristics of the corresponding cell type.

### The databases for predicting transcription factor binding sites and potential protein-protein interactions

The AnimalTFDB 3.0 and hTFtarget databases were employed in this study to forecast potential transcription factor binding sites situated on the CCNB1 promoter region. Additionally, STRING was utilized to analyze and predict potential protein-protein interactions involving SNRPB. These databases facilitated a more comprehensive understanding of the functional roles of SNRPB and CCNB1 in the progression of HCC.

### Gene expression profiling

Gene expression profiling using the Illumina NextSeq 500 platform (Illumina, USA) was applied to compare the transcriptomic differences between HCC tumor samples (*n* = 10) and their corresponding adjacent normal samples (*n* = 10), as well as between shCtrl and shSNRPB groups of SK-HEP-1 cells (*n* = 3). In detail, we extracted Total RNA with the RNeasy Mini Kit (Qiagen, Germany) and its concentration was measured with the NanoDrop 2000 spectrophotometer (Thermo Fisher Scientific, USA). The quality of the RNA was evaluated via the the Agilent 2100 Bioanalyzer (Agilent Technologies, USA) to ensure that it met the required quality standards (RNA Integrity Number ≥ 7). RNA was then converted into complementary DNA (cDNA) via the TruSeq RNA Sample Prep Kit (Illumina, USA), followed by amplification and fragmentation. The resulting cDNA libraries were hybridized to the Illumina HumanHT-12 V4 BeadChip (Illumina, USA). Following this, the arrays were washed, stained, and scanned using the Illumina iScan System (Illumina, USA). To derive gene expression profiles, the raw data were analyzed via Illumina GenomeStudio software (Illumina, USA). The differentially expressed genes (DEGs) were pinpointed through a comparison of expression levels between the two groups, with a fold change ≥ 2.0 and statistical significance (*P* ≤ 0.05) as the selection criteria. The biological significance of the DEGs were enriched with pathway in gene ontology (GO) and KEGG databases to gain an overview of biological significance. In addition, hierarchical clustering was used for visualization and interpretation of the differential expression patterns among the samples.

### Cell culture

The cell lines used in the study are as follows: HL-7702, SK-HEP-1, HCCLM3, Huh-7, and Hep3B. HL-7702 cells were grown in RPMI-1640 medium (Gibco, USA) with 10% fetal bovine serum (FBS) (Gibco, USA). SK-HEP-1 and HCCLM3 cells were cultured in DMEM medium (Gibco, USA) with 10% high-quality FBS (Gibco, USA). Huh-7 and Hep3B cells were kept in MEM medium (Gibco, USA) supplemented with 10% FBS. All cells were cultured at 37 °C in a humidified environment with 5% CO₂. Culture media and supplements were refreshed every 2–3 days, and the cells were passaged once they reached approximately 80–90% confluence.

### Lentiviral construction and cell transduction

For the SNRPB knockdown experiments, three specific shRNAs targeting SNRPB were constructed: SNRPB-27,102, SNRPB-27,103, and SNRPB-27,104. The target sequences for these shRNAs are listed in Table S1. Lentiviral vectors containing these shRNAs were generated using standard protocols, and the viruses were packaged in HEK-293T cells. In brief, HEK-293T cells were transfected with the shRNA constructs and packaging plasmids (psPAX2 and pMD2.G) using Lipofectamine 2000 (Thermo Fisher Scientific, USA). After 48 h, the viral supernatants were collected, filtered, and used to infect SK-HEP-1 and HCCLM3 cells. Successfully infected cells were cultivated in 1 µg/mL puromycin (Sigma-Aldrich, USA) for 7–10 days. For overexpression of SNRPB, a plasmid encoding the full-length human SNRPB gene was used (Shanghai Yibeirui Biomedical Science and Technology, China). The plasmid was transfected into SK-HEP-1 and HCCLM3 cells using Lipofectamine 2000 reagent (Thermo Fisher Scientific, USA). Similarly, for CCNB1 knockdown, lentiviral vectors containing specific shRNAs targeting CCNB1 were constructed and transfected into SK-HEP-1 and HCCLM3 cells using the same protocol. After transfection, cells were cultured in complete medium for 48–72 h to allow for protein expression or knockdown, and subsequently subjected to various assays to evaluate the impact of SNRPB overexpression or CCNB1 knockdown on HCC cell behaviors.

### Clinical sample collection and immunohistochemistry staining

In this study, the tissue microarrays were acquired from Shanghai Yibeirui Biomedical Science and Technology (China), with catalog numbers YBR-HlivHep88-M006 (for SNRPB) and YBR-HlivHep89-M006 (for CCNB1). All clinical samples were obtained and studied with the approval of 050432-4-2108*, with informed consent obtained from the patients to collect their tissue samples for study.

For immunohistochemical (IHC) staining, tissue sections underwent deparaffinized, rehydrated, and subjected to antigen retrieval with a sodium citrate buffer (pH 6.0). Following the 10-minute blockage of endogenous peroxidase activity with 3% hydrogen peroxide, the slides were incubated with primary antibodies (as listed in Table S2) overnight at 4 °C. The next day, slides were incubated with appropriate secondary antibodies for 1 h at room temperature, followed by detection using the DAB substrate (Dako, #K3468, Denmark). The final procedures included counterstaining the slides with hematoxylin, dehydrating them, and then mounting them. Subsequent evaluation of IHC staining intensity and distribution was independently conducted by two pathologists. The scales to evaluate stain intensity were as follows: 0 (no staining), 1 (weak), 2 (moderate), and 3 (strong), while the percentage of positively stained cells was classified as 0 (< 10%), 1 (10-25%), 2 (26-50%), 3 (51-75%), and 4 (> 75%). To calculate the final staining score, the intensity score was multiplied by the percentage score, yielding values between 0 and 12. The median IHC score of all samples was utilized to separate the samples into high and low expression groups.

### Quantitative polymerase chain reaction (qPCR)

The extraction of total RNA from the cultured cells or tissue samples was carried out using TRIzol reagent (Sigma, #T9424-100 m). With a NanoDrop ND-2000 (Thermo Scientific), the RNA quantity and quality were assessed in the study. To synthesize cDNA, 1 µg of total RNA was reverse transcribed with the Hiscript QRT Supermix for qPCR (+ gDNA WIPER) (Vazyme, #R123-01). The reaction was carried out at 42 °C for 30 min, followed by 85 °C for 5 s to deactivate the reverse transcriptase. For quantitative PCR, the AceQ qPCR SYBR Green Master Mix (Vazyme, #Q111-02) on a QuantStudio 5 Real-Time PCR System (Applied Biosystems) was employed in the study. The reaction mixture (20 µL) consisted of 10 µL of SYBR Green Master Mix, 1 µL of cDNA, and 1 µL of each primer (forward and reverse) with 7 µL of nuclease-free water. The cycling conditions for the PCR included an initial denaturation at 95 °C for 30 s, followed by 40 cycles of amplification (95 °C for 10 s and 60 °C for 30 s). With the temperature ranging from 60 °C to 95 °C in 0.5 °C increments, we conducted melting curve analysis to confirm the specificity of the amplification. Gene expression levels were normalized to the internal reference gene, GAPDH, and calculated in the 2^−ΔΔCt^ method. Table S3 showed the primer sequences used for qPCR.

### Western blotting (WB)

For cell lysate preparation, RIPA buffer was employed along with protease inhibitors to maintain protein integrity in the process. The BCA Protein Assay Kit (HyClone-Pierce, #23225, USA) was utilized to determine protein concentrations. The protein samples (20–30 µg) were separated by SDS-PAGE and subsequently transferred to PVDF membranes to facilitate immunoblotting. The PVDF membranes, after being blocked with 5% non-fat dry milk in TBS-T for 1 h at room temperature, were subjected to an overnight incubation at 4 °C with the primary antibodies for targeted antigen recognition (Table S2). The membranes were then washed with TBS-T and incubated with appropriate HRP-conjugated secondary antibodies for 1 h at room temperature. The visualization of immunoreactive proteins was achieved via Immobilon Western Chemiluminescent HRP Substrate (Millipore, #RPN2232, USA) and detected by Chemiluminescence Imaging System (GE Healthcare, UK). ImageJ software (NIH, USA) were employed to quantify protein bands. The expression of target proteins was normalized to GAPDH, and the relative protein quantities were computed based on the densitometric values.

### Cell counting Kit-8 (CCK-8) assay

Cell Counting Kit-8 (CCK-8, Sigma, #96992, USA) was performed for the evaluation of cell viability. In brief, we seeded cells in 96-well plates (Corning, #CLS3596, USA) at a density of 5 × 10^3^ cells per well in 100 µL of culture medium. Following incubation periods of 24 h, 48 h, or 72 h, 10 µL of CCK-8 solution was added to each well, and the cells were incubated for an additional 2 h at 37 °C. The microplate reader (Tecan Infinite, #M2009PR, Switzerland) was used to measure the absorbance at 450 nm. The cell viability was calculated by comparing the absorbance values of experimental groups to the control group, with data normalized to the control.

### Colony formation assay

In this study, seeding of SK-HEP-1 and HCCLM3 cells was performed in 6-well plates for the colony formation assay, with each well receiving a population of 500-1,000 cells. Following this, the plates were placed in a 5% CO2 environment at 37 °C for 10–14 days to allow colony formation. The culture medium was refreshed every 2–3 days to maintain cell viability and growth. After the incubation period, the colonies were treated with 4% paraformaldehyde for fixation and then stained using crystal violet. Observation and quantification of the colonies were conducted under a light microscope, with only those surpassing the threshold of 50 cells considered positive.

### Flow cytometry analysis

In this study, the Annexin V-FITC/PI double staining method was employed via flow cytometry to assess the apoptosis of cells. Briefly, cells were harvested, washed with PBS, and resuspended in binding buffer. Then, 5 µL of Annexin V-FITC and 5 µL of propidium iodide (PI) solution were added to each sample. Following a 15-minute incubation period at room temperature in darkness, the flow cytometric was assessed with a flow cytometer (Millipore, USA) to detect Annexin V-positive and PI-positive cells. The apoptotic cells were identified based on the presence of Annexin V staining, and the late apoptotic/necrotic cells were identified by PI positivity.

### Wound-healing assay

A scratch wound healing assay was considered to be a simple and effective approach for the assessment of cell migration. Briefly, cells were cultured in 6-well plates and permitted to reach confluency. Subsequently, a uniform scratch was generated with a 96 Wounding Replicator (#VP408FH, VP Scientific, USA). Following this, the cells were rinsed with PBS and then incubated in a serum-free medium. With images captured at 0 h and 24 h with a light microscope (Olympus, Japan), the area of the wound was quantified through ImageJ software (National Institutes of Health, USA). The percentage of wound closure was calculated based on the measured areas to determine the migration rate.

### Transwell migration assay

The assessment of cell migration was conducted using a Transwell chamber (Corning). In brief, cells were serum-starved for 24 h before being seeded into the upper chamber at a density of 1 × 10^5^ cells in 200 µL serum-free medium. The lower chamber was filled with 600 µL medium containing 10% fetal bovine serum (FBS) to act as a chemoattractant. The incubation period took place at 37 °C for 24 h. After this time, the cells that had migrated to the bottom surface were fixed using 4% paraformaldehyde for 15 min. Subsequently, they were stained with crystal violet for 20 min to allow visualization. Counting the number of cells in five randomly selected fields under a microscope (Olympus), the quantification of migrated cells was performed.

### Subcutaneous tumor xenograft in nude mice

BALB/c nude mice (4 weeks old) under specific pathogen-free conditions were obtained from Charles River (China). All procedures involving animal experiments were carried out in accordance with institutional ethical guidelines, and approval was obtained from FUSCC-IACUC-S2023-0398. To establish subcutaneous tumors, 4 × 10^6^ cells were resuspended in 100 µL of PBS and injected into the right flank of each mouse. Tumor growth was monitored at indicated intervals, and tumor volume was measured with calipers with the formula: volume = 0.5 × length × width^2^. The experimental duration was 35 days. Finally, the mice were euthanized, and their tumors were subsequently analyzed.

### Chromatin Immunoprecipitation (ChIP) assay

Cells were exposed to a 1% formaldehyde solution at room temperature for 10 min to facilitate the capture of protein-DNA crosslinks. After washing and lysing the cells, chromatin was fragmented through sonication to achieve smaller DNA pieces. The chromatin was then incubated with anti-FOXM1 antibody or control IgG (negative control) overnight at 4 °C. The antibody-protein-DNA complexes were pulled down using ChIP-Grade Protein G Agarose Beads (CST, #9006, USA) and washed several times with high-salt buffer to remove non-specific bindings. To analyze FOXM1 binding to the CCNB1 promoter, the enriched DNA was purified and subjected to quantitative PCR (qPCR) to quantify the amplification of CCNB1 promoter regions. The primers for qPCR were detailed in Table S3. The relative binding of FOXM1 to the CCNB1 promoter was normalized to the input and IgG control samples.

### Dual-luciferase reporter assay

In the study, we performed the dual-luciferase reporter assay to investigate the transcriptional activity of FOXM1 on the CCNB1 promoter. The wild-type CCNB1 promoter or mutant constructs were cloned into the pGL3-basic luciferase reporter vector (Promega, USA). Mutations were introduced into the CCNB1 promoter at specific positions: Mut1 (from − 702 to -690, sequence aaaacaaacaaaa mutated to cggcaccgacgg), Mut2 (from − 90 to -75, sequence CCCTTCCCATTGGCGG mutated to AATAACCGTTTCCTTT), and Mut3 (from − 58 to -43, sequence CCCTCCTTATTGGCCT mutated to CTTAACCGCCTTCTTT) to assess the impact of FOXM1 binding on promoter activity.

Cells were co-transfected with the reporter plasmids and FOXM1 overexpression or control vectors using Lipofectamine 3000 (Thermo Fisher Scientific, USA). Following a 48 h incubation period, the cells were lysed to extract the necessary components for analysis. Dual-Luciferase Reporter Assay System (Promega, USA) was performed to assess the luciferase activity. For accuracy, the activity of firefly luciferase was normalized against that of Renilla luciferase, which served as a reference for transfection efficiency. The results are presented as relative luciferase activity, allowing for straightforward comparisons. Furthermore, the influence of FOXM1 on the CCNB1 promoter was analyzed by comparing the responses between wild-type and mutant constructs.

### Co-immunoprecipitation (Co-IP) assay

The investigation of the protein-protein interaction between SNRPB and FOXM1 was conducted using Co-IP assays. Cell lysates were prepared using RIPA buffer (Beyotime, China). Immunoprecipitation was performed by incubating 1 mg of total protein with 2 µg of SNRPB antibody (refer to Table S2 for antibody details) overnight at 4 °C, followed by incubation with Protein A + G magnetic beads (Beyotime, China) for 2 h at 4 °C. To eliminate nonspecific binding, the beads underwent three washes with RIPA buffer. Subsequently, the immunoprecipitated complexes were eluted by boiling in SDS-PAGE loading buffer. For the eluted proteins, WB analysis was conducted to detect the presence of FOXM1. Enhanced chemiluminescence with Immobilon Western Chemiluminescent HRP Substrate (Millipore, USA) was utilized for protein visualization, followed by imaging on a GE imaging system (GE Healthcare, USA).

### Detection of lipid metabolism indicators

Triglycerides, total cholesterol, and free fatty acids levels were measured using commercially available assay kit. In brief, cell lysates were collected and processed as per the instructions provided with the respective kits. The triglyceride assay kit (Solarbio, #BC0625, China) was utilized to measure TG levels, a total cholesterol assay kit (Solarbio, #BC1985, China) was employed to quantify TC level, and a free fatty acid assay kit (Solarbio, #BC0595, China) was applied to determine FFA levels. The concentrations of TG, TC, and FFA were calculated based on standard curves provided by the kits.

### IC50 determination of cisplatin

To determine the half-maximal inhibitory concentration (IC50) of cisplatin on the tested cells, a cell viability assay was conducted using the CCK-8 method. Briefly, cells were treated with different concentrations of cisplatin (10 µM, 30 µM, 100 µM, 300 µM, 1000 µM, 3000 µM) for 24 h. After incubation, CCK-8 solution was added to each well according to the instructions. The calculation IC50 value included plotting the percentage of cell viability versus cisplatin concentration and determining the concentration that inhibited cell viability by 50%.

### Statistical analysis

GraphPad Prism 8.0 (GraphPad Software, USA) and SPSS 22.0 (IBM, USA) were utilized for statistical analysis in the study. The unpaired two-tailed t-test was employed for comparing two sets of data. To compare with multiple groups, a one-way analysis of variance (ANOVA) was conducted, followed by Tukey’s post-hoc test to ascertain specific group differences. Kaplan-Meier method was employed for survival analysis, and the log-rank test was conducted to assess the significance of survival differences. Relationships between pathological parameters and gene expression levels were examined using Pearson’s or Spearman’s correlation coefficients based on the nature of the data. A statistically significant p-value was considered to be less than 0.05.

## Results

### The tumor microenvironment (TME) of HCC

**Fig. 1:**
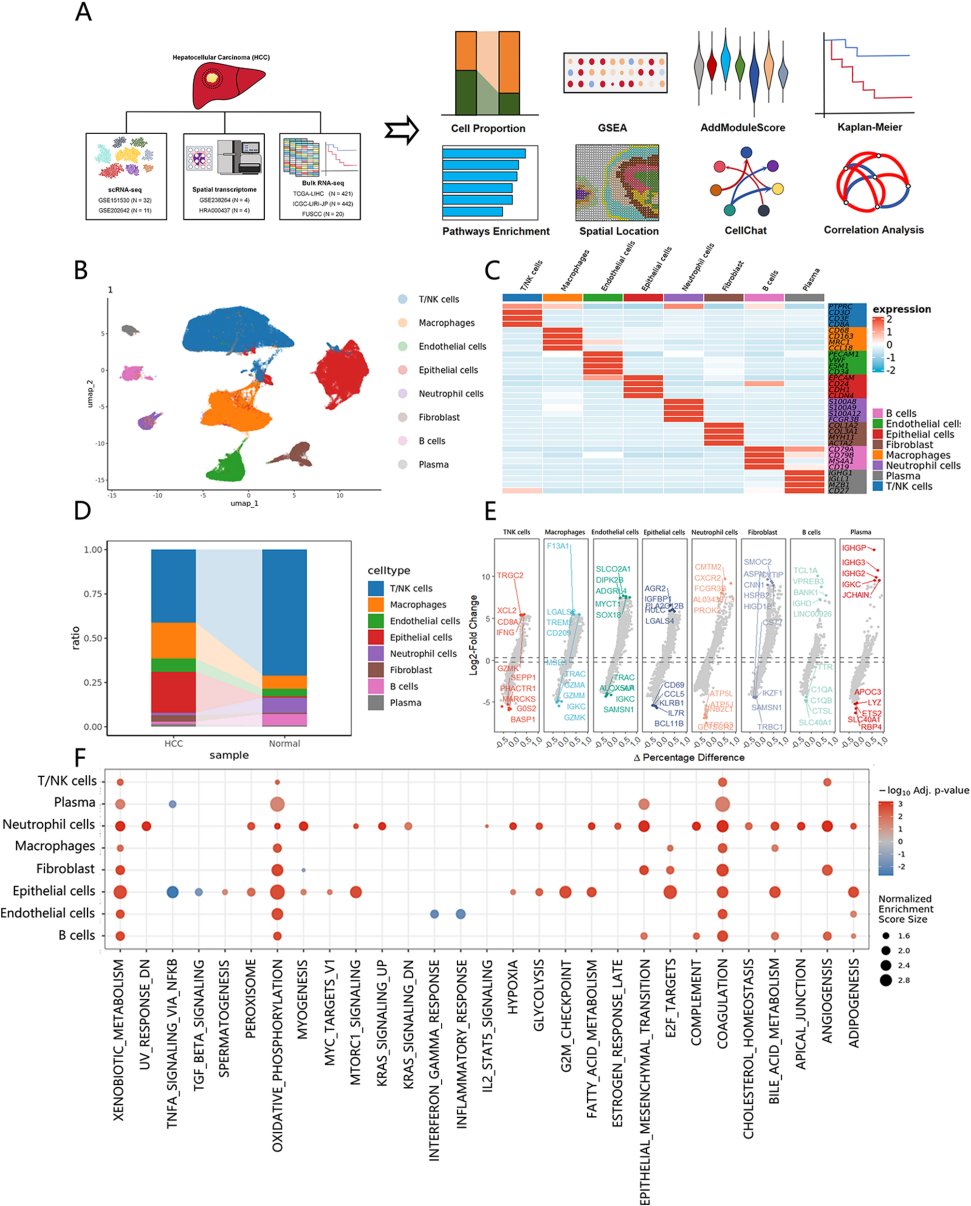
Fig. 1

The workflow of bioinformatics analysis was summarized in Fig. 1A. To investigate the heterogeneity between the primary tumor and the normal tissue in HCC at the single-cell level, we conducted an integrated analysis of two datasets derived from GSE151530 (*n* = 32) [21] and GSE202642 (*n* = 11) [22]. After QC, a total of 129,401 cells were included in the subsequent analysis. Performing dimensionality reduction analysis, our analysis revealed 26 cellular clusters within the sample, each characterized by the expression patterns of specific marker genes. It allowed for the classification of these clusters into eight major cell types: nine T/NK cells clusters, seven Epithelial cells clusters, four Macrophages clusters, two B cell clusters, and others clusters (Fig. 1B and C). With some marker genes like ALB [28] and ALDOB [29]we distinguished the malignant cells from immune and stromal cells, which were predominantly mixed with epithelial cells (Figure S1A). Subsequently, to investigate compositional heterogeneity between tumor and normal tissue groups, we elucidated the proportions of each major cell type. In detail, HCC exhibited a significant reduction of T/NK cells, neutrophils, and B cells and enrichment of macrophages compared to normal tissues (Fig. 1D), indicating the presence of a macrophage-mediated immunosuppressive microenvironment in HCC. Within our defined epithelial cells, the expression of oncogenes like IGFBP1 30 and HULC [31] was significantly up-regulated (Fig. 1E), further demonstrating that epithelial cells and malignant cells cluster together in the same subset due to their homogeneity.

To explore the regulatory changes of cells in the TME, we summarized the enrichment of common pathways between tumor and normal tissues using the GSEA algorithm (Fig. 1F). Consistent with findings from another study [32], the oxidative phosphorylation pathway was highly upregulated in TME, indicating that the proliferation and survival of some subtypes of tumors were not solely reliant on glycolysis, but oxidative phosphorylation also played a significant role. Given the liver’s distinctive metabolic characteristics, the fatty and bile acid metabolism exerted significant effects on the TME.

### The role of spliceosome prognostic genes in HCC

Aiming to unravel the role of the spliceosome in HCC advancement, we extracted 127 spliceosome-associated genes from the KEGG database and performed univariate Cox regression analysis in TCGA and ICGC cohorts (Table S4). Notably, a total of 15 genes (*EIF4A3*,* LSM2*,* LSM4*,* LSM5*,* PPIL1*,* PRPF19*,* SF3A3*,* SNRPA1*,* SNRPB*,* SNRPB2*,* SNRPD1*,* SNRPF*,* SNRPG*,* SRSF9*,* TXNL4A*) demonstrated consistent associations with poor prognosis across both cohorts (*P* < 0.05, HR > 1), which were regarded as spliceosome prognostic genes for subsequent analyses. Furthermore, examining the differential expression levels of identified genes in tumor and normal tissues facilitated evaluating their potential as drug targets. It was noteworthy that these genes all showed higher expression levels in tumor tissues, suggesting their potential as key oncogenes driving the development of cancer (Figure S1B).

**Fig. 2:**
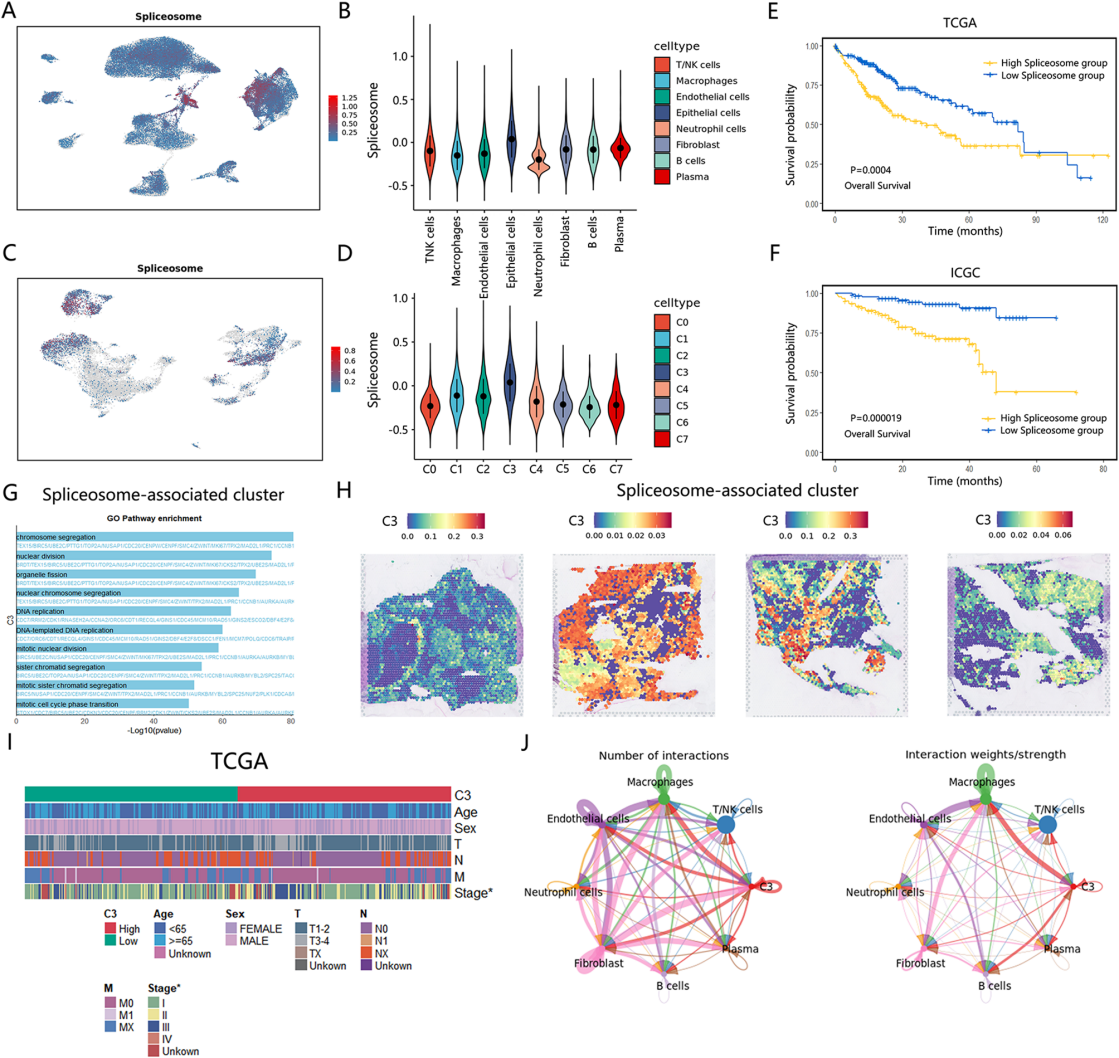
The role of spliceosome-associated cluster in HCC. (**A**) The UMAP plots of global cell type illustrating the enrichment scores of spliceosome prognostic genes in HCC patients. (**B**) Violin plot of enrichment scores of spliceosome prognostic genes in global cell type in HCC patients. (**C**) The UMAP plots of epithelial cells illustrating the enrichment scores of spliceosome prognostic genes in HCC patients. (**D**) Violin plot of enrichment scores of spliceosome prognostic genes in epithelial cells in HCC patients. (**E**) Survival analysis based on TCGA cohort showed that elevated scores of spliceosome prognostic genes were associated with poor prognosis in HCC patients. (**F**) Survival analysis based on ICGC cohort showed that elevated scores of spliceosome prognostic genes were associated with poor prognosis in HCC patients. (**G**) The result of enrichment analysis for specifically expressed genes of C3 cluster. (**H**) The spatial distribution of C3 cluster in HCC determined by ST data (GSE238264). (**I**) The heatmap showing the association between C3 cluster and clinical factors in TCGA cohort. (**J**) The communication network between C3 cluster and other cell types in HCC patients

Based on scores of the spliceosome prognostic genes on Gene Set Variation Analysis (GSVA), we stratified patients in TCGA and ICGC into high and low spliceosome groups by median for subsequent survival analysis (Fig. 2E and F). The result indicated poorer survival in HCC patients classified as the high spliceosome group. Using Addmodulescore algorithm, the expression of spliceosome-associated prognostic pathway in cells were scored and observed a significant enrichment in epithelial cells (Fig. 2A and B). Due to the mixture of malignant cells and epithelial cells, additional dimensionality reduction clustering of epithelial cells is essential for pinpointing the precise cell subtype where the spliceosome gene set demonstrated elevated expression levels. The epithelial cluster was reannotated as 7 subclusters. Among them, C3 cluster exhibited a significant upregulation of the spliceosome prognostic genes (Fig. 2C and D). The heatmap and score of inferCNV demonstrated that this cluster was indeed comprised of malignant cells (Figure S2A and S2B). Further analysis revealed that C3 cluster widely distributed in different tumor samples rather than in normal tissues, confirming the above results. Above all, C3 cluster was regarded as the spliceosome-associated tumor cluster to conduct a further exploration.

The results of enrichment analysis based on specifically expressed genes of C3 cluster revealed that this cluster had a strong chromosome segregation and DNA replication in comparison to other subclusters (Fig. 2G). This suggested that C3 cluster had a strong proliferative capacity. Utilizing scRNA-seq data as a reference, the integrated mapping approach in Seurat was used to accurately pinpoint the spatial localization of C3 cluster on ST sections (Fig. 2H and S2C). This cluster was founded to widely distributed in the tumor region, further validating it as a tumor cell subset. Given the limited sample size in our scRNA-seq dataset, Bisque, [33]a deconvolution algorithm, was employed to estimate the abundance of the C3 subpopulation within the TCGA and ICGC cohorts. In TCGA cohorts, this cluster was found to be related to the clinical stages of patients (Fig. 2I). With the expression of paired receptors and ligands in different cell types analyzed by Cellchat, the intricate landscape of cellular communication within TME was illustrated(Fig. 2J). A significant interactive relationship between the C3 cluster and macrophages were shown in HCC. Macrophages, pivotal immune cells within TME, were considered to encompass powerful immunomodulatory effects, potentially facilitating or suppressing antitumor immune responses. Previous research indicated that crosstalks between tumor cells and macrophages reshaped the immunosuppressive TME. Tumor cells could use the “Don’t eat me” signal, such as CD47 34, SIRPG [35] PD-1^36^ and CD24 37, to escape recognition and phagocytosis by macrophages, which were significant contributors to the recurrence, metastasis, and therapeutic resistance of various cancers.

Given the significant biological value of the spliceosome in HCC progression, a more in-depth analysis of underlying mechanisms is imperative to comprehensively understand its functional significance in cancer.

### Identification of SNRPB as a key regulator in HCC

**Fig. 3:**
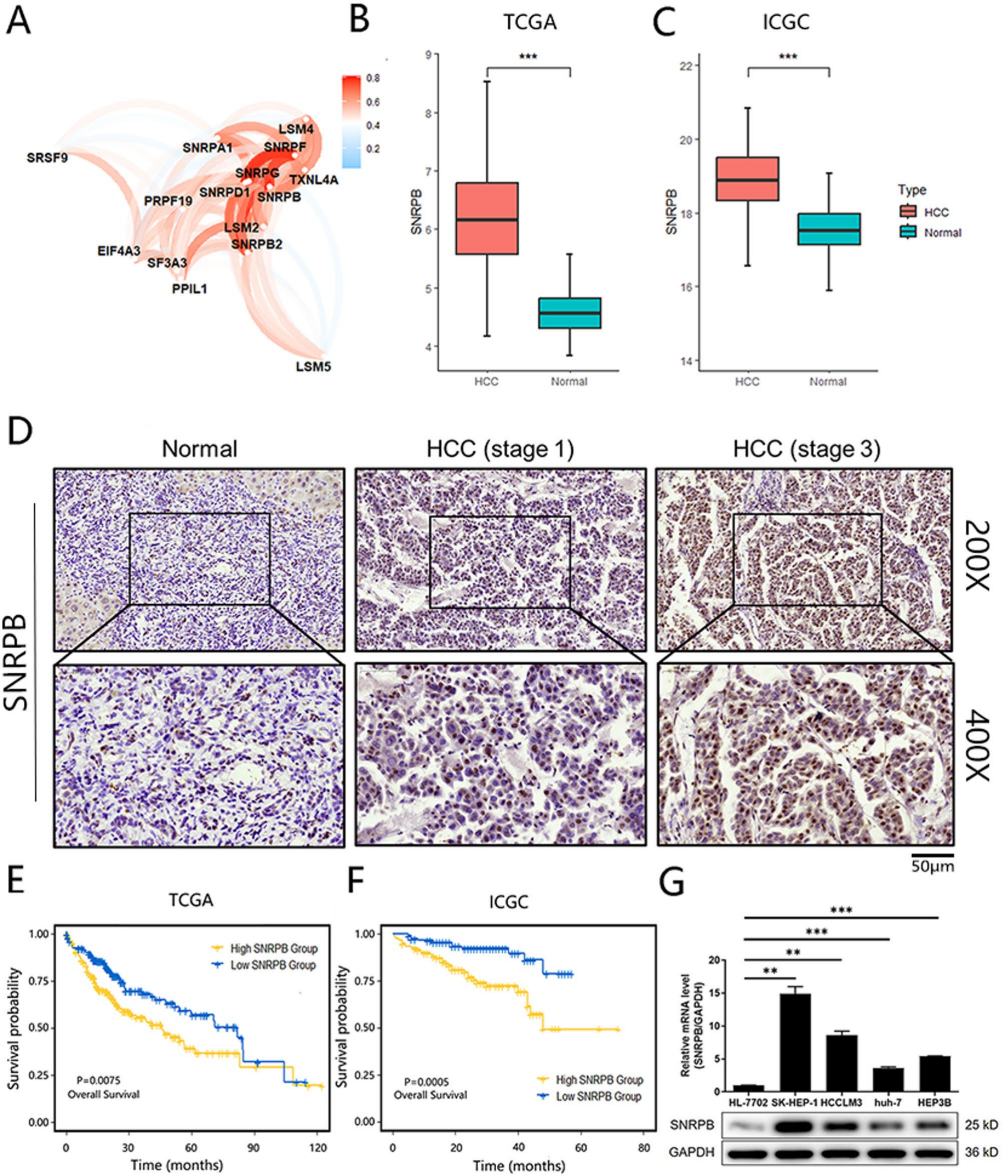
Identification of SNRPB as a key regulator in HCC. (**A**) The plot illustrating the correlation among the 15 identified spliceosome-related genes. (**B**) The box plot showing elevated SNRPB expression in HCC compared to normal liver tissues in TCGA cohorts. (**C**) The box plot showing elevated SNRPB expression in HCC compared to normal liver tissues in ICGC cohorts. (**D**) IHC analysis revealed significant upregulation of SNRPB expression in HCC tissues compared to normal tissues. (**E**) Survival analysis based on TCGA cohort showed that elevated SNRPB expression was associated with poor prognosis in HCC patients. (**F**) Survival analysis based on ICGC cohort showed that elevated SNRPB expression was associated with poor prognosis in HCC patients. (**G**) Endogenous SNRPB expression was compared in normal liver cells (HL-7702) and HCC cell lines, with significantly higher levels observed in HCC cells. Data were drawn as mean ± SD (*n* ≥ 3). * *P* < 0.05, ** *P* < 0.01, *** *P* < 0.001

To begin with, a correlation analysis among these identified genes suggested that SNRPB exhibited significant correlations with multiple genes, highlighting its crucial role in the gene regulatory networks (Fig. 3A). Next, a series of experiments targeting SNRPB were proceeded to gain a more profound comprehension of its underlying mechanism in the HCC progression. Firstly, the bulk RNA-seq and PCR was evaluated in a local cohort (FUSCC) including 10 HCC and 10 adjacent normal tissues, suggesting that SNRPB mRNA levels were significantly higher in tumor tissues (Figure S3A and S3B). Similarly, results of bulk RNA-seq from the five large clinical cohorts confirmed elevated SNRPB expression in HCC compared with normal tissues (Fig. 3B and C, Figure S3B). IHC performed on tissue microarray further validated the expression pattern of SNRPB. The IHC staining results (Fig. 3D) and corresponding statistical analyses (Table 1) showed that SNRPB expression exhibited a significant upregulation in HCC tissues as opposed to normal tissues. Additionally, higher SNRPB expression levels were positively associated with advanced pathological stage and T stage, suggesting a link between SNRPB expression and tumor malignancy (Fig. 3D; Table 2 and S5). Survival analysis conducted using both TCGA and ICGC database (Fig. 3E and F) revealed that elevated SNRPB expression was significantly associated with poor prognosis, further highlighting its clinical relevance in HCC progression. The same result is also verified in FUSCC (Figure S3C). Finally, endogenous expression of SNRPB was examined in normal liver cells (HL-7702) and multiple HCC cell lines. The significant increase in SNRPB expression observed in HCC cell lines, in comparison to normal liver cells, indicated a potential association between elevated SNRPB levels and HCC pathogenesis (Fig. 3G). The collective evidence implies a critical involvement of SNRPB in the advancement of HCC.

**Table 1:** Expression patterns of SNRPB in HCC tissues and normal tissues revealed in immunohistochemistry analysis

SNRPB expression	Tumor tissue		Normal tissue	
	Cases	Percentage	Cases	Percentage
Low	36	46.8%	60	92.3%
High	41	53.2%	5	7.7%

*P* < 0.001

**Table 2:** Relationship between SNRPB expression and tumor characteristics in patients with HCC

Features	No. of patients	SNRPB expression		*P* value
		low	high	
All patients	77	36	41	
Age (years)				0.555
< 59	37	16	21	
≥ 59	40	20	20	
Gender				0.244
Male	64	28	36	
Female	13	8	5	
Grade				0.198
II	41	22	19	
III	36	14	22	
Tumor size				0.055
≤ 5.2 cm	38	22	16	
> 5.2 cm	39	14	25	
Stage				0.020
1	24	15	9	
2	37	17	20	
3	15	4	11	
4	1	0	1	
T				0.038
T1	24	15	9	
T2	38	17	21	
T3	5	0	5	
T4	10	4	6	

### SNRPB knockdown inhibits HCC progression in vitro and in vivo

**Fig. 4:**
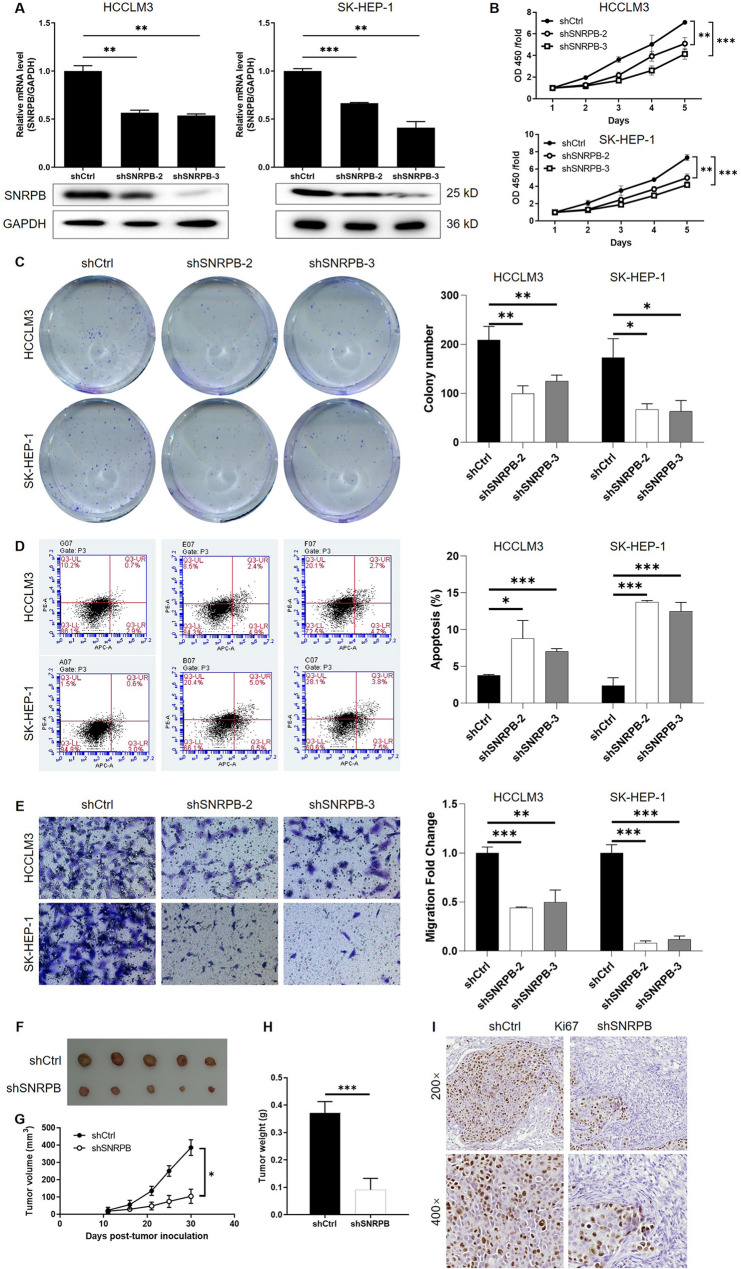
Functional analysis of SNRPB knockdown in HCC cells. (**A**) Validation of SNRPB knockdown efficiency in SK-HEP-1 and HCCLM3 cells at both mRNA and protein levels using two effective shRNAs (shSNRPB-2 and shSNRPB-3). (**B**) CCK-8 assay demonstrates the inhibitory effects of SNRPB knockdown on HCC cell proliferation in SK-HEP-1 and HCCLM3 cells. (**C**) Colony formation assay shows that SNRPB knockdown significantly suppresses the clonogenic capacity of HCC cells. (**D**) Flow cytometry analysis reveals that SNRPB knockdown significantly increases apoptosis rates in SK-HEP-1 and HCCLM3 cells. (**E**) Transwell assay demonstrates that silencing SNRPB significantly reduces the migratory capacity of HCC cells. (**F**) The pictures taken from the excised xenografts of indicated experimental groups. (**G**) Tumor volume measurements in subcutaneous xenograft tumor models showing the effects of SNRPB knockdown on tumor growth. (**H**) Tumor weight analysis at the time of sacrifice in xenograft models. (**I**) IHC staining of tumor tissues showing Ki67 expression levels. Data were drawn as mean ± SD (*n* ≥ 3). * *P* < 0.05, ** *P* < 0.01, *** *P* < 0.001

To investigate the functional role of SNRPB in HCC, SK-HEP-1 and HCCLM3 cells, which exhibited relatively high endogenous SNRPB expression, were selected for loss-of-function studies. Three shRNAs targeting SNRPB were designed, and qPCR analysis identified shSNRPB-2 and shSNRPB-3 as the most effective constructs for SNRPB knockdown (Figure S4). The knockdown efficiencies were further validated in HCCLM3 and SK-HEP-1 cells at both mRNA and protein levels (Fig. 4A). Functional assays were then performed to assess the impact of SNRPB knockdown on HCC cell phenotypes. The results from the CCK-8 assay demonstrated a significant decrease in the proliferation of HCC cells upon SNRPB silencing in comparison to the shCtrl group (Fig. 4B). Colony formation assays further confirmed that SNRPB knockdown markedly suppressed the clonogenic capacity of HCC cells (Fig. 4C). Flow cytometry was used to analyze apoptosis, facilitating investigating the impact of SNRPB on cell survival. SNRPB knockdown significantly increased apoptosis rates in both HCCLM3 and SK-HEP-1 cells (Fig. 4D). The experimental assessment of cell migration following SNRPB knockdown, using both wound-healing (Figure S5A) and transwell (Fig. 4E) assays, consistently revealed a marked decline in the migratory capacity of HCC cells. Consistent with these findings, analysis of epithelial-mesenchymal transition (EMT) markers showed that SNRPB knockdown resulted in elevated expression of E-cadherin and reduced levels of N-cadherin, Vimentin, and Snail (Figure S5B), further supporting its role in promoting HCC cell migration. Moreover, HCCLM3 with SNRPB knockdown formed significantly smaller xenografts than that without SNRPB knockdown (Fig. 4F and H), which also showed lower expression of Ki67, a proliferation marker (Fig. 4I). Together, these results indicates that SNRPB knockdown suppresses HCC progression in vitro and in vivo.

### SNRPB regulates CCNB1 expression *via* FOXM1-mediated transcriptional activation

**Fig. 5:**
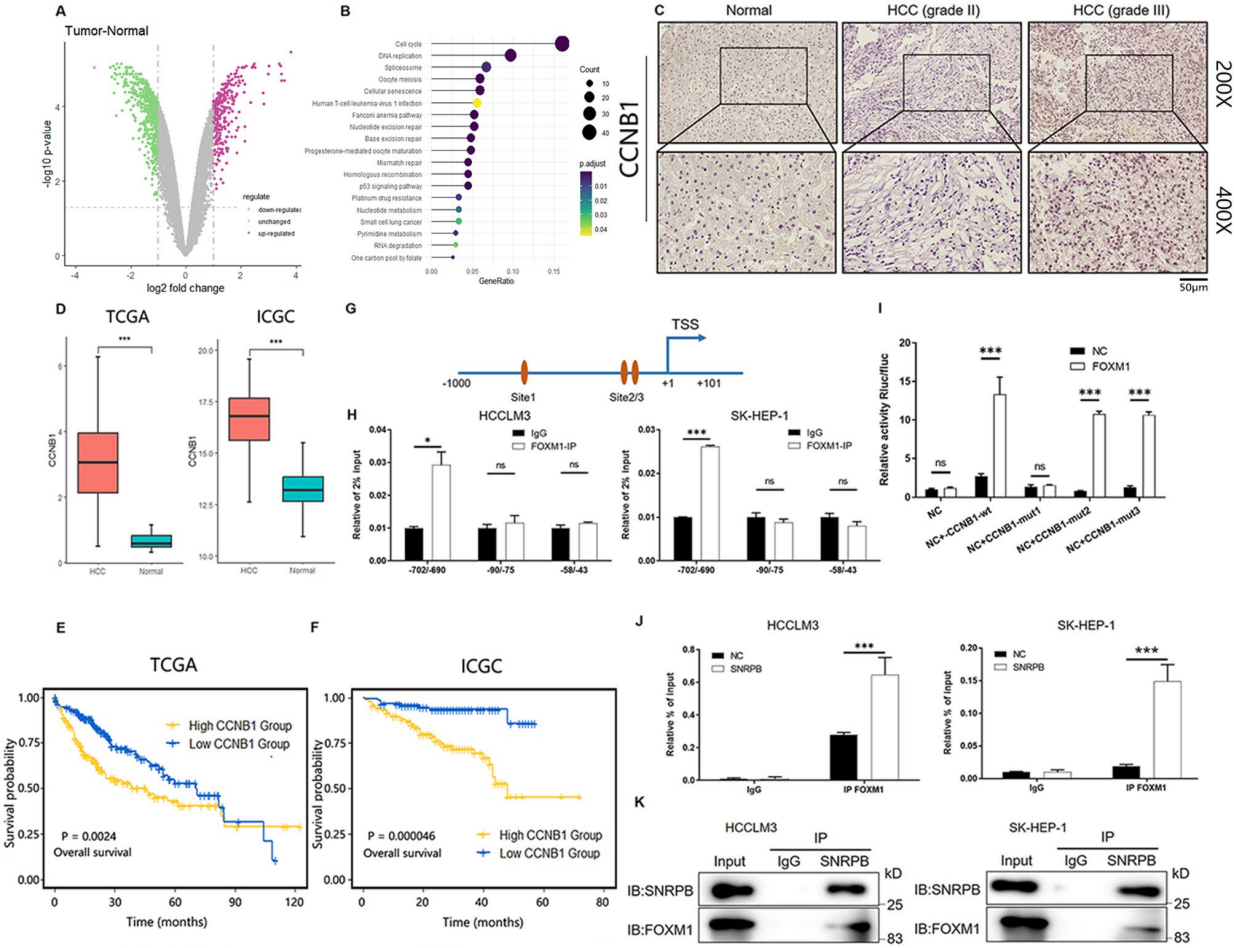
Mechanistic analysis of SNRPB-mediated regulation of CCNB1 expression in HCC. (**A**) Volcano plot illustrating the significantly upregulated and downregulated genes identified in SK-HEP-1 cells following SNRPB knockdown. (**B**) The results of KEGG pathway enrichment analysis on the significantly downregulated genes. (**C**) IHC analysis of tissue microarray showing elevated CCNB1 expression in HCC tissues compared to adjacent normal tissues. (**D**) The box plot showing elevated CCNB1 expression in HCC compared to normal liver tissues in TCGA and ICGC cohorts. (**E**) Survival analysis based on TCGA cohort showing negative correlation between CCNB1 expression and overall survival in HCC patients. (**F**) Survival analysis based on ICGC cohort showing negative correlation between CCNB1 expression and overall survival in HCC patients. (**G**) Identification of FOXM1-binding sites in the CCNB1 promoter using bioinformatics tools. (**H**) ChIP assay demonstrating significant FOXM1 enrichment at Site 1 of the CCNB1 promoter in SK-HEP-1 cells. (**I**) Dual-luciferase reporter assay confirming that mutations at Site 1 (CCNB1-mut1) abolish FOXM1-mediated transcriptional activation of CCNB1. (**J**) ChIP assay showing enhanced FOXM1 enrichment at the CCNB1 promoter in SK-HEP-1 cells overexpressing SNRPB. (**K**) Co-IP assay demonstrating a protein-protein interaction between SNRPB and FOXM1. Data were drawn as mean ± SD (*n* ≥ 3). * *P* < 0.05, ** *P* < 0.01, *** *P* < 0.001

To uncover the downstream mechanism by which SNRPB modulated HCC advancement, gene expression profiling was performed using SK-HEP-1 cells transfected with shSNRPB-2 or shCtrl. A total of 286 significantly upregulated (*P* < 0.05 and logFC > 1) and 572 significantly downregulated (*P* < 0.05 and logFC < -1) genes were identified (Fig. 5A). Subsequently, KEGG pathway enrichment analysis was performed on the significantly downregulated genes, revealing that the cell cycle was markedly enriched (Fig. 5B). Therefore, we focused on the role of cell cycle genes in downregulated genes. By integrating genes in cell cycle (KEGG) with downregulated genes, we pinpointed 34 genes that were significantly downregulated in gene expression profiling. Through univariate Cox regression analysis of them, we identified the five genes with the smallest *P*-value in each of the two cohorts and defined them as top five genes (Table S6). Among them, CCNB1, a cell cycle regulator, was involved as one of top five genes in both TCGA and ICGC cohorts, and showed a high HR among them. Furthermore, previous literature suggested that SNRPB showed a significant co-expression with CCNB1^38^. Based on these results, we speculated that CCNB1 may play an important role in the downstream mechanism of SNRPB in HCC proliferation.

To investigate the regulatory mechanism of SNRPB on CCNB1, we directed our attention to TFs, which are pivotal mediators in controlling gene expression. By integrating known transcription factors (AnimalTFDB 3.0) with downregulated genes, we pinpointed 25 TFs that were significantly downregulated in gene expression profiling (Figure S6A and Table S7). Subsequent protein interaction network analysis (STRING) revealed that three of them (FOXM1, SSRP1, TP53) exhibited interactions with SNRPB (Figure S6B). Finally, through hTFtarget, FOXM1 was ascertained to regulate CCNB1 as a TF.

IHC analysis of tissue microarray revealed elevated CCNB1 expression in tumor tissues in comparison to adjacent normal tissues, with higher expression levels significantly associated with advanced pathological grade (Fig. 5C, Tables S8–S10). Analysis of TCGA and ICGC databases supported these results, revealing a markedly elevated expression of CCNB1 in HCC tissues in comparison to normal tissues (Fig. 5D). Furthermore, the strong link between CCNB1 expression and poor prognosis was consistently evident across both cohort, highlighting the potential utility of CCNB1 as a valuable prognostic indicator in HCC (Fig. 5E and F). In addition, the above results were validated in the FUSCC cohort as well (Figure S6E and S6F).

To understand how SNRPB regulates CCNB1 expression, we first verified the reduced CCNB1 protein levels in SNRPB knockdown SK-HEP-1 and HCCLM3 cells (Figure S6C). Additionally, it was found that the endogenous expression patterns of CCNB1 and SNRPB exhibited similarities in HCC cell lines and normal liver cells (Figure S6D). Using bioinformatic tools (AnimalTFDB 3.0 and hTFtarget), three potential FOXM1-binding sites were identified in the CCNB1 promoter (Fig. 5G). ChIP assays showed significant FOXM1 enrichment at Site 1 (-702~-690), but not at Sites 2 or 3 (Fig. 5H). This was further validated by dual-luciferase reporter assays, where mutations at Site 1 (CCNB1-mut1) abolished FOXM1-mediated transcriptional activation, confirming that FOXM1 primarily binds to Site 1 to regulate CCNB1 expression (Fig. 5I). Further ChIP experiments revealed that SNRPB overexpression enhanced FOXM1 enrichment at the CCNB1 promoter, suggesting that SNRPB promoted CCNB1 expression by augmenting FOXM1-mediated transcriptional activation (Fig. 5J). Co-IP assays confirmed a protein-protein interaction between SNRPB and FOXM1, providing a mechanistic basis for SNRPB to facilitate FOXM1-dependent CCNB1 transcription (Fig. 5K). Together, these findings indicated that SNRPB promotes CCNB1 expression through FOXM1-mediated transcriptional activation, contributing to HCC progression.

### The functional role of the SNRPB/CCNB1 axis in regulating HCC progression

**Fig. 6:**
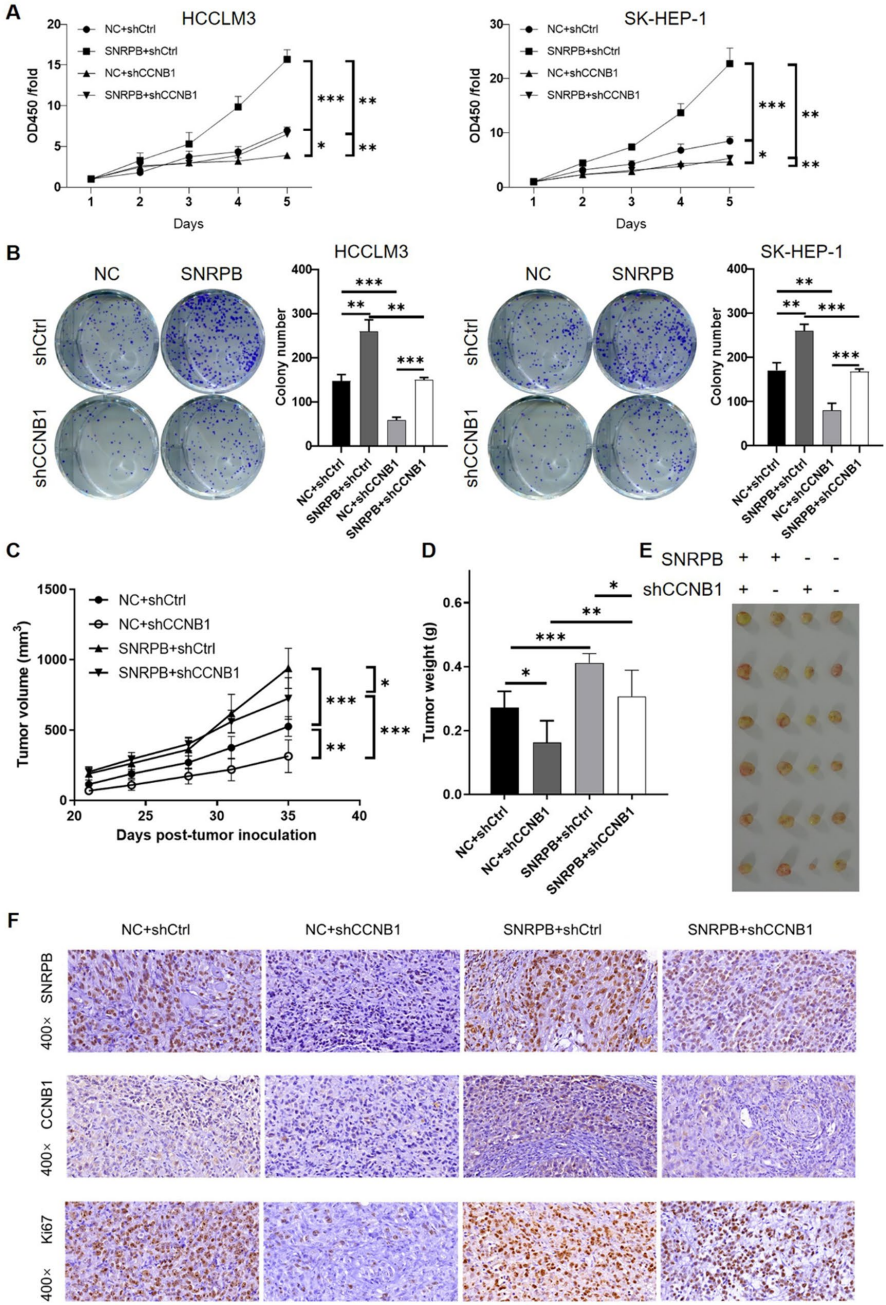
The functional role of CCNB1 in mediating the effects of SNRPB on HCC progression. (**A**) CCK-8 assay assessing the proliferation of HCC cells in four experimental groups: control (NC + shCtrl), SNRPB overexpression (SNRPB + shCtrl), CCNB1 knockdown (NC + shCCNB1), and combined SNRPB overexpression and CCNB1 knockdown (SNRPB + shCCNB1), highlighting the restoration of proliferation upon CCNB1 knockdown in SNRPB-overexpressing cells. (**B**) Colony formation assay showing the impact of SNRPB overexpression and CCNB1 knockdown on HCC cell colony formation, with a focus on the restoration of clonogenic capacity in the combined treatment group. (**C**) Tumor volume measurements in subcutaneous xenograft tumor models showing the effects of SNRPB overexpression and CCNB1 knockdown on tumor growth, emphasizing the partial reversal of enhanced tumor growth by CCNB1 knockdown in SNRPB-overexpressing tumors. (**D**) Tumor weight analysis at the time of sacrifice in xenograft models, highlighting the partial reversal of increased tumor weight due to CCNB1 knockdown in SNRPB-overexpressing tumors. (**E**) The pictures taken from the excised xenografts of indicated experimental groups. (**F**) IHC staining of tumor tissues showing SNRPB, CCNB1 and Ki67 expression levels, with a focus on the restoration of Ki67 expression in the combined treatment group, indicating the reversal of the effects of SNRPB overexpression. Data were drawn as mean ± SD (*n* ≥ 3). * *P* < 0.05, ** *P* < 0.01, *** *P* < 0.001

To explore the functional role of CCNB1 in mediating the effect of SNRPB on HCC progression, we generated four experimental groups: control (NC + shCtrl), SNRPB overexpression (SNRPB + shCtrl), CCNB1 knockdown (NC + shCCNB1), and a combination of SNRPB overexpression and CCNB1 knockdown (SNRPB + shCCNB1). Assessment of cell proliferation through the CCK-8 assay suggested that the overexpression of SNRPB led to an enhanced proliferation of HCC cells, while CCNB1 knockdown significantly inhibited cell proliferation. Notably, when CCNB1 was knocked down in SNRPB overexpressing cells, the proliferative effects induced by SNRPB was largely restored, indicating that CCNB1 mediates the effects of SNRPB in HCC cell proliferation (Fig. 6A). In addition, results from clonogenic assays showed similar results as CCK-8 assay, further confirming that CCNB1 is crucial for SNRPB-induced tumorigenic effects in HCC cells (Fig. 6B). To further evaluate the SNRPB/CCNB1 axis in vivo, we established subcutaneous xenograft tumor models. Tumor volume measurements revealed that SNRPB overexpression promoted tumor growth, while CCNB1 knockdown significantly inhibited tumor growth. Importantly, the combination of SNRPB overexpression and CCNB1 knockdown resulted in a partial reversal of the enhanced tumor growth induced by SNRPB overexpression (Fig. 6C). Consistently, tumor weights recorded at the time of sacrifice were in line with the cell proliferation results. The SNRPB overexpression group exhibited significantly heavier tumors, while CCNB1 knockdown led to a reduction in tumor weight. The combined treatment group showed an intermediate tumor weight, suggesting that CCNB1 knockdown partly mitigates the tumor-promoting effect of SNRPB overexpression (Fig. 6D and E). IHC staining of tumor tissues further revealed that SNRPB overexpression correlated with higher Ki67 expression, indicating enhanced cell proliferation. In contrast, CCNB1 knockdown led to a significant reduction in Ki67 expression. The combined treatment group exhibited Ki67 levels that were intermediate between the SNRPB overexpression and CCNB1 knockdown groups (Fig. 6F). These results collectively demonstrated that SNRPB promoted HCC cell proliferation and tumor growth through the upregulation of CCNB1. That is to say, the SNRPB/CCNB1 axis was found to be of great importance in HCC progression.

### SNRPB modulates lipid metabolism in HCC cells

**Fig. 7:**
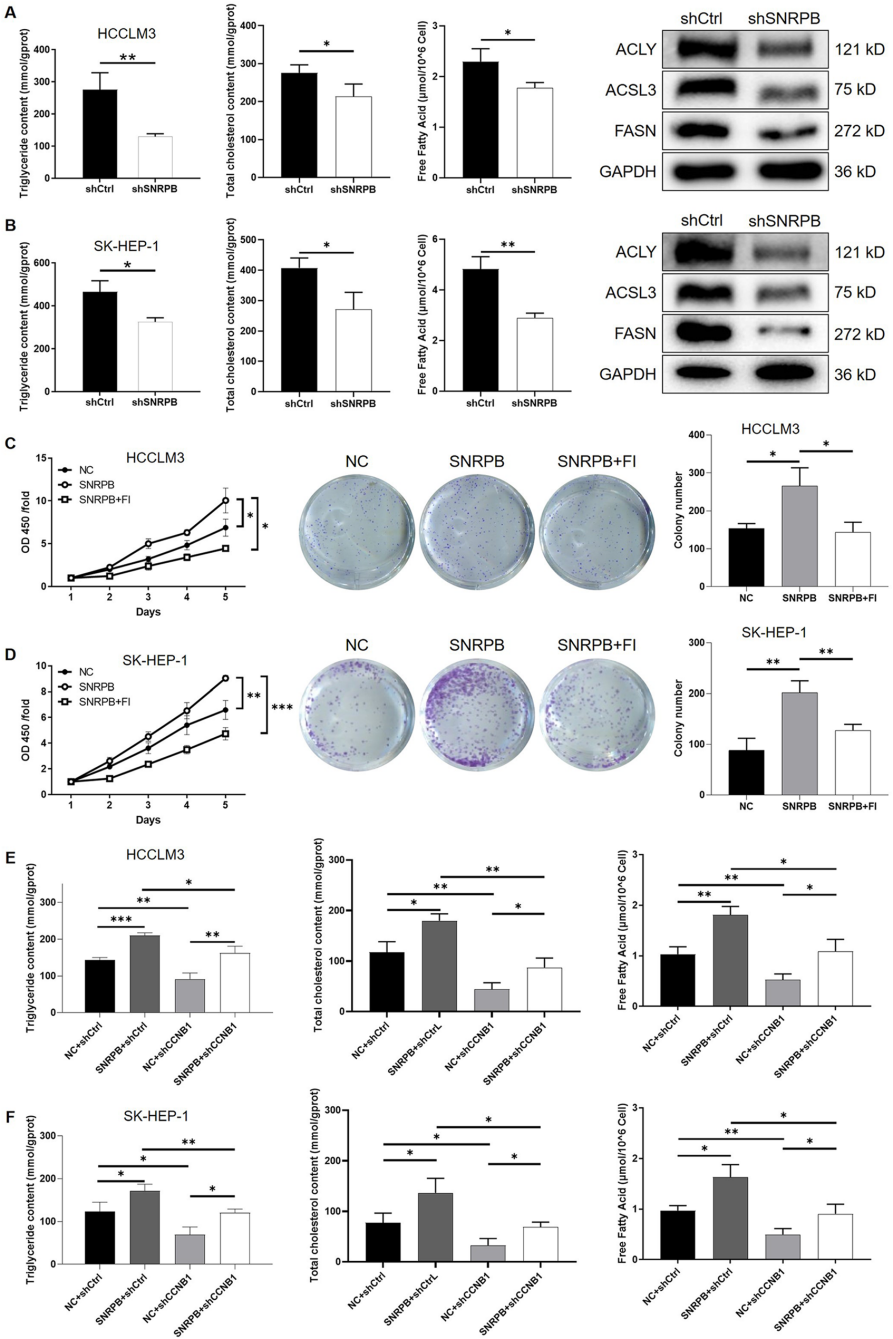
Investigation of SNRPB and CCNB1 in regulating lipid metabolism in HCC cells. (**A**) Triglyceride, total cholesterol, and free fatty acid levels were detected in HCCLM3 cells following SNRPB knockdown, showing a reduction in lipid metabolism markers. (**B**) Triglyceride, total cholesterol, and free fatty acid levels were detected in SK-HEP-1 cells following SNRPB knockdown, showing a similar reduction in lipid metabolism markers. (**C**) The CCK-8 assay and colony formation assay in HCCLM3 cells showing the reversal of proliferative and clonogenic advantages by ACLY inhibition. (**D**) The CCK-8 assay and colony formation assay in SK-HEP-1 cells showing the reversal of proliferative and clonogenic advantages by ACLY inhibition. (**E**) Triglyceride, total cholesterol, and free fatty acid levels were detected in HCCLM3 cells, demonstrating the effect of SNRPB overexpression and CCNB1 knockdown on lipid metabolism, with partial reversal of lipid metabolism enhancement by CCNB1 knockdown. (**F**) Triglyceride, total cholesterol, and free fatty acid levels were detected in SK-HEP-1 cells, showing the interplay between SNRPB overexpression and CCNB1 knockdown in regulating lipid metabolism. Data were drawn as mean ± SD (*n* ≥ 3). * *P* < 0.05, ** *P* < 0.01, *** *P* < 0.001

Lipid metabolism reprogramming is recognized as a critical feature of tumor cells. Previous studies have suggested a potential linkage between SNRPB and lipid metabolism reprogramming in lung carcinoma [39]. Hence, we extended our focus to examine the correlation between SNRPB and lipid metabolism within HCC. Our results revealed that knockdown of SNRPB significantly reduced the levels of triglycerides, total cholesterol, and free fatty acids in HCC cells, while also downregulating the expression of key lipid metabolism-related proteins, including ACLY, FASN, and ACSL3, in both HCCLM3 (Fig. 7A) and SK-HEP-1 (Fig. 7B) cells. Furthermore, to examine the functional role of SNRPB in lipid metabolism, we co-expressed SNRPB with the ACLY inhibitor SB204990 (FI), which reversed the lipid metabolism enhancement induced by SNRPB overexpression. This reversal was evident in the decreased levels of triglycerides, total cholesterol, and free fatty acids in both HCCLM3 (Figure S7A) and SK-HEP-1 (Figure S7B) cells. Additionally, FI treatment alleviated the proliferative and clonogenic advantages of SNRPB overexpression, further confirming the role of SNRPB in regulating lipid metabolism in HCC cells (Fig. 7C for HCCLM3, Fig. 7D for SK-HEP-1). Moreover, rescue experiments showed that SNRPB overexpression enhanced lipid metabolism, while CCNB1 knockdown inhibited these effects. Notably, CCNB1 knockdown partially reversed the lipid metabolism enhancement induced by SNRPB overexpression in both HCCLM3 (Fig. 7E) and SK-HEP-1 (Fig. 7F) cells, highlighting the interplay between SNRPB and CCNB1 in regulating lipid metabolism in HCC.

### SNRPB modulates cisplatin sensitivity in HCC cells *via* lipid metabolism and CCNB1

**Fig. 8:**
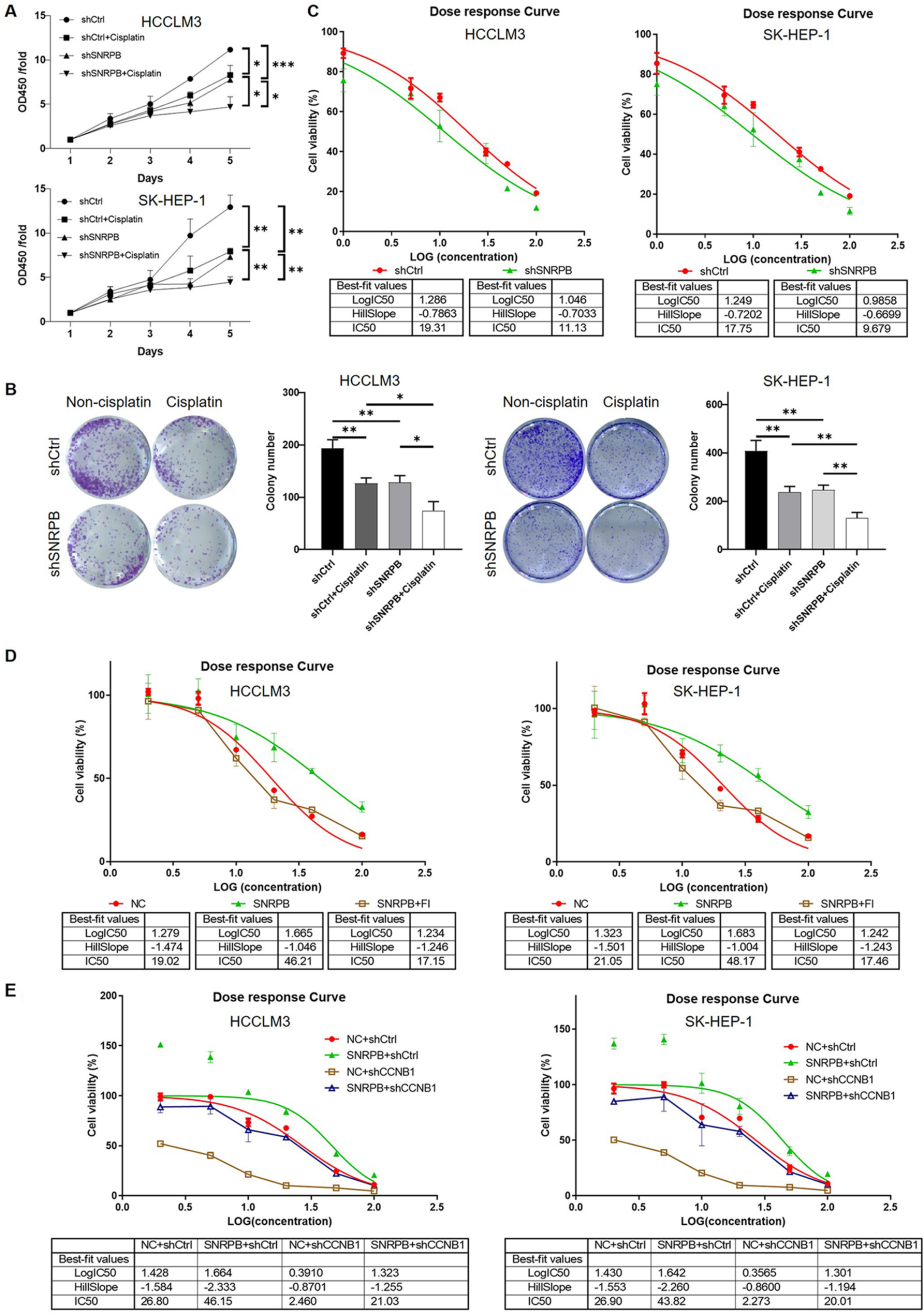
The role of SNRPB in regulating cisplatin sensitivity in HCC cells through lipid metabolism and CCNB1 modulation. (**A**) CCK-8 assay assessing the proliferation of HCC cells treated with cisplatin, SNRPB knockdown, or both, showing the synergistic effect of combined treatments on cell proliferation inhibition. (**B**) Colony formation assay showing the impact of cisplatin and SNRPB knockdown on clonogenic ability of HCC cells, highlighting the synergistic inhibition of colony formation by combined treatments. (**C**) IC50 values of cisplatin in shCtrl and shSNRPB HCC cell lines (HCCLM3 and SK-HEP-1), showing increased cisplatin sensitivity upon SNRPB knockdown. (**D**) IC50 values of cisplatin in HCC cells treated with ACLY inhibitor (FI) in combination with SNRPB overexpression, indicating the restoration of cisplatin sensitivity through lipid metabolism inhibition. (**E**) IC50 values of cisplatin in HCC cells with SNRPB overexpression and CCNB1 knockdown, demonstrating the modulation of cisplatin sensitivity by the SNRPB/CCNB1 axis. Data were drawn as mean ± SD (*n* ≥ 3). * *P* < 0.05, ** *P* < 0.01, *** *P* < 0.001

In light of the potential link between lipid metabolism reprogramming and drug sensitivity in cancer cells, we further proceeded to investigate the impact of SNRPB on the regulation of cisplatin sensitivity in HCC cells. Initially, we observed that both SNRPB knockdown and cisplatin treatment similarly inhibited HCC cell proliferation, with a synergistic effect when both treatments were combined (Fig. 8A for CCK-8 assay, Fig. 8B for colony formation assay). In order to further quantify the influence of SNRPB on cisplatin sensitivity, we measured the IC50 values of cisplatin in both shCtrl and shSNRPB HCC cell lines (HCCLM3 and SK-HEP-1). It was observed that the knockdown of SNRPB significantly lowered the IC50 values of cisplatin, indicating an increased sensitivity to cisplatin in these cells (Fig. 8C). We next explored the involvement of lipid metabolism reprogramming and CCNB1 in SNRPB-related modulation of cisplatin sensitivity in HCC cells. Treatment with the ACLY inhibitor FI was found to restore the reduced IC50 induced by SNRPB overexpression, suggesting a role for lipid metabolism in regulating cisplatin sensitivity (Fig. 8D). Additionally, a rescue experiment revealed that SNRPB overexpression reduced cisplatin sensitivity in HCC cells, whereas CCNB1 knockdown increased sensitivity. Notably, the combination of SNRPB overexpression and CCNB1 knockdown alleviated the effect of SNRPB overexpression on cisplatin sensitivity (Fig. 8E), further implicating the SNRPB/CCNB1 axis in modulating drug response.

## Discussion

As a core component of the spliceosome, SNRPB plays an essential role in pre-mRNA splicing and transcript maturation. Evidence has been presented that SNRPB was implicated in different biological processes including cell cycle regulation and metabolic pathways. For instance, Knill et al. demonstrated that SNRPB deficiency suppressed osteogenesis while enhancing chondrogenesis in mesenchymal stem cells, likely through modulation of the Wnt/β-catenin and BMP signaling pathways [15]. Furthermore, Alam et al. revealed that SNRPB was critical for craniofacial development, with its loss-of-function linked to splicing defects and dysregulation of p53 pathway components [14]. These findings underscore the significance of SNRPB in normal developmental processes. As mentioned before, SNRPB has been linked to cerebro-costo-mandibular syndrome (CCMS), a congenital skeletal dysplasia. Mechanistic investigations revealed that SNRPB mutations lead to alternative splicing defects, particularly in genes involved in craniofacial and skeletal development, such as Smad2 and Rere [14]. These studies emphasize the tissue-specific impact of spliceosomal dysfunction associated with SNRPB. The oncogenic potential of SNRPB has been extensively studied in numerous malignant tumors. SNRPB was identified as upregulated in 28 out of 33 cancer types analyzed, including cervical, ovarian, and thyroid carcinomas [38]. In cervical cancer, SNRPB knockdown was reported to suppress cell proliferation and promote apoptosis through its interaction with modulation of p53 activity [18]. Similarly, in ovarian cancer, SNRPB promoted tumor progression by regulating DNA replication and homologous recombination pathways [40]. Its role in HCC has also been reported. Li et al. demonstrated that SNRPB facilitated HCC progression by modulating alternative splicing events, promoting the formation of AKT3-204 and LDHA-220 isoforms, which activated the Akt signaling pathway and aerobic glycolysis, respectively [20]. These studies collectively supported SNRPB as a key oncogenic driver in multiple cancer types. Our research shed new light on the oncogenic role SNRPB played in the advancement of HCC. Using gene expression profiling, we found that SNRPB exhibited a significant upregulation in tumor tissues, in comparison to adjacent normal tissues. Functional analyses demonstrated that the knockdown of SNRPB inhibited HCC cell phenotypes in vitro and suppressed tumor growth in vivo significantly. These results emphasize the oncogenic potential of SNRPB in promoting HCC progression. Interestingly, discrepancies existed between our findings and certain reports in non-HCC contexts. For example, while SNRPB promotes HCC progression, its role in non-cancerous conditions such as CCMS involves dysregulated splicing and developmental defects rather than proliferative advantages. This divergence may stem from tissue-specific regulatory mechanisms or differential downstream targets. Our study further investigated the interplay between SNRPB, lipid metabolism, and cisplatin sensitivity. Lipid metabolism reprogramming is a hallmark of cancer [41–45]and we observed that SNRPB knockdown reduced triglyceride, cholesterol, and free fatty acid levels in HCC cells by downregulating key metabolic enzymes such as ACLY, FASN, and ACSL3 46, 47. Importantly, treatment with the ACLY inhibitor SB204990 reversed the enhanced lipid metabolism and proliferative effects induced by SNRPB overexpression, underscoring the role of lipid metabolism in SNRPB-mediated oncogenesis. A variety of published studies have demonstrated the association between lipid metabolism reprogramming of cancer cells with their drug resistance [48, 49]. Our data revealed that SNRPB modulated cisplatin sensitivity in HCC cells *via* lipid metabolism. Knockdown of SNRPB enhanced cisplatin sensitivity, as evidenced by reduced IC50 values, while overexpression reduced sensitivity.

CCNB1 is a crucial regulator of the cell cycle, specifically involved in the G2/M transition. As a cyclin, CCNB1 activates cyclin-dependent kinases, facilitating the progression of cells through mitosis [50–52]. CCNB1 has been identified as tumor-promotor in multiple tumors. Evidence suggests that aberrant CCNB1 expression is correlated with unfavorable survival outcomes in various cancers, including ovarian carcinoma, prostate cancer, and lung adenocarcinoma [53–56]. These findings suggests that elevated levels of CCNB1 can contribute to tumor progression by promoting cell proliferation and inhibiting cell cycle checkpoints. Specifically, in ovarian carcinoma, CCNB1 overexpression enhanced cell proliferation, migration, and invasion, while its knockdown inhibited these processes [54]. Furthermore, in prostate cancer, CCNB1 expression was linked to poor prognosis, with knockdown of CCNB1 inhibiting cell proliferation and metastasis [55]. The relevance of CCNB1 in HCC has also been established. Elevated CCNB1 expression is observed in HCC tissues, where it correlates with poor overall survival and advanced pathological grade [57]. Furthermore, CCNB1 overexpression is associated with increased DNA replication and cell cycle progression, contributing to tumorigenesis in HCC. Importantly, recent research has proposed that CCNB1 played a role in the resistance of different cancer types, such as prostate cancer, to chemotherapy [58]. These suggested that CCNB1 may play a role in mediating drug resistance, an aspect of tumor biology that warrants further investigation.

Some limitations need to be acknowledged. Firstly, the reliability of results may be constrained by a limited size of the local cohort, subsequent studies will include more samples through multi-center collaborations to improve the robustness of the result. **S**econdly, although shRNA-mediated gene suppression reflects the essentiality of SNRPB, single-direction interventions may lead to an incomplete portrayal of the functional maps of SNRPB. Overexpression experiments should be carefully included in the subsequent research to make a comprehensive judgment. Thirdly, due to the expensive costs of sequencing and limited availability of samples, our current analysis has been conducted on a limited sample size, which may limit statistical power. It’s essential to expand the sample size to strengthen the robustness of results in subsequent research. Fourthly, Although splicesome inhibitors have therapeutic potential, their nonspecific effects still need to be optimized, and recently developed CRISPR-based approaches are more suitable for subsequent targeted studies. Lastly, due to limited time and resources, functional redundancy or compensatory mechanisms from paralogs like SNRPD1 or SNRPE were not fully demonstrated. Future studies should examine their roles to clarify this compensatory relationship.

In this study, we offered some new insights into the regulation of CCNB1 in HCC, particularly as a downstream mediator of SNRPB. We found that SNRPB, a key splicing factor, regulated CCNB1 expression *via* FOXM1-mediated transcriptional activation. This regulatory mechanism underscored the pivotal role of the SNRPB/CCNB1 axis in driving HCC progression. Moreover, the overexpression of SNRPB enhanced cell proliferation and tumor growth, which was significantly mitigated by CCNB1 knockdown, confirming that CCNB1 mediated the tumor-promoting effects of SNRPB in HCC. Furthermore, our findings suggested that the SNRPB/CCNB1 axis was not only crucial for cell proliferation but also for lipid metabolism in HCC cells. We observed that CCNB1 knockdown partially reversed the metabolic changes induced by SNRPB overexpression. In addition to regulating proliferation and metabolism, the SNRPB/CCNB1 axis also modulated cisplatin sensitivity of HCC cells. Our data revealed that SNRPB knockdown sensitized HCC cells to cisplatin, while CCNB1 knockdown enhanced this effect. Therefore, it was demonstrated that targeting the SNRPB/CCNB1 axis may increase the efficacy of chemotherapy in HCC, a potential strategy for overcoming drug resistance in this malignancy.

In conclusion, this study revealed that SNRPB promoted HCC progression by modulating the FOXM1-CCNB1 axis and lipid metabolism, indicating its potential as a therapeutic target to augment chemotherapy sensitivity in HCC.

## Electronic supplementary material

Below is the link to the electronic supplementary material.

Supplementary Material 1

Supplementary Material 2

## Data Availability

The information of FUSCC cohort can be available from the corresponding author upon reasonable request. Apart from the local cohort (FUSCC cohort), the transcriptome data and clinical information incorporated in this study are all publicly accessible, with details described in the section “Materials and methods”.
