# Supplementary material for: SNRPB/CCNB1 axis promotes hepatocellular carcinoma progression and cisplatin resistance through enhancing lipid metabolism reprogramming
Source: J Exp Clin Cancer Res. 2025 Jul 18;44:211. doi: 10.1186/s13046-025-03463-y (PMC12273286; doi:10.1186/s13046-025-03463-y)
Supplement: Supplementary file 1 — Supplementary Material 1 [file 13046_2025_3463_MOESM1_ESM.docx]

**Table S1. The target sequences and shRNA sequences**

| Gene | No. | Target sequence (5'-3') |
| --- | --- | --- |
| SNRPB | SNRPB-27102 | TTCAGCTAATACCCAAGGAAA |
| SNRPB | SNRPB-27103 | TGGCTTTAAAGACCATGAAGA |
| SNRPB | SNRPB-27104 | CTTGAGACAGCTACAAGGATT |

**Table S2. Antibodies used in WB and IHC**

| Primary antibodies | Dilution in WB | Source species | Company | Catalog No. |
| --- | --- | --- | --- | --- |
| CCNB1 | 1:3000 | Rabbit | Abcam | ab32053 |
| GAPDH | 1:30000 | Mouse | Proteintech | 60004-1-lg |
| SNRPB | 1:1000 | Rabbit | Proteintech | 16807-1-AP |
| N-Cadherin | 1:1000 | Rabbit | Bioss | bs-1172R |
| E-Cadherin | 1:3000 | Rabbit | Proteintech | 20874-1-AP |
| Vimentin | 1:2000 | Rabbit | Proteintech | 10366-1-AP |
| SNAIL | 1:1000 | Rabbit | sigma | SAB1306281 |
| CDCA5 | 1:500 | Rabbit | Abcam | ab192237 |
| RAD51AP1 | 1:1000 | Rabbit | Abcam | ab101321 |
| ACLY | 1:1000 | Mouse | Proteintech | 67166-1-Ig |
| FASN | 1:5000 | Rabbit | Proteintech | 10624-2-AP |
| ACSL3 | 1:5000 | Rabbit | Abclonal | A22085 |
| FOXM1 | 1:2000 | Rabbit | Proteintech | 13147-1-AP |
|  |  |  |  |  |
| Primary antibodies | Dilution in IHC | Source species | Company | Catalog No. |
| SNRPB | 1:50 | Mouse | Thermo | MA5-13449 |
| CCNB1 | 1:100 | Rabbit | Bioss | bs-0572R |
|  |  |  |  |  |
| Secondary antibody | Dilution |  | Company | Catalog No. |
| HRP Goat Anti-Rabbit IgG | 1:3000 |  | Beyotime | A0208 |
| HRP Goat Anti-Mouse IgG | 1:3000 |  | Beyotime | A0216 |
| HRP Goat Anti-Rabbit IgG | 1:200 |  | Abcam | Ab111909 |

**Table S3. Primers used in qPCR**

| Gene | Forward primer sequence (5’-3’) | Reverse primer sequence (5’-3’) |
| --- | --- | --- |
| GAPDH | TGACTTCAACAGCGACACCCA | CACCCTGTTGCTGTAGCCAAA |
| SNRPB | GGATCTTCATTGGCACCTTCA | CTCTTCCCTTTCTGCTTGTTTG |
| CCNB1 | AAACTTTGGTCTGGGTCGGC | TGCTGCAATTTGAGAAGGAGG |
| KIF4A | CCATACCCATTGACTAACTAT | ACCCCTTCCTCTTCACTTTC |
| RNASEH2A | ATCGCAAATGCTGCACTACG | GGTCACATCCTGGAAGAAAGG |
| UBE2S | CTACCAGATGGCACTTACACCC | GCAAAGATACAGCCAACACTCC |
| KIF11 | ACCACCTGATGAATATCTGTACTACGC | TGCAGGGTCTGCAATGCTACG |
| CCNB1 -F1 (ChIP) | TTGGCAACTGGTCTAAGTATGA | TTCACAACACTGATTGTCCCTT |
| CCNB1 -F2 (ChIP) | AATGGGAAGGGAGTGAGTGC | GAGAAGAGCCAGCCTAGCCT |
| CCNB1 -F3 (ChIP) | CCACGAACAGGCCAATAAGGA | GACCAGCCAAGGACCTACAC |
|  |  |  |

**Table S4. The risk genes identified by univariate Cox regression analyses for spliceosome-associated genes in TCGA and ICGC cohorts**

| Database | Gene | Coef | HR (95% CI) | P. value | Type |
| --- | --- | --- | --- | --- | --- |
| TCGA | SF3A3 | 0.88 | 2.4 (1.7-3.4) | 2.1e-07 | Risk |
| TCGA | RBM17 | 0.75 | 2.1 (1.6-2.9) | 1.4e-06 | Risk |
| TCGA | SF3B4 | 0.56 | 1.8 (1.4-2.2) | 1.5e-06 | Risk |
| TCGA | PRPF19 | 0.96 | 2.6 (1.8-3.9) | 1.8e-06 | Risk |
| TCGA | NCBP2 | 0.73 | 2.1 (1.5-2.8) | 4.3e-06 | Risk |
| TCGA | EFTUD2 | 0.7 | 2 (1.5-2.7) | 5.2e-06 | Risk |
| TCGA | PRPF38A | 0.75 | 2.1 (1.5-2.9) | 5.9e-06 | Risk |
| TCGA | USP39 | 0.78 | 2.2 (1.5-3.1) | 2.7e-05 | Risk |
| TCGA | HNRNPM | 0.75 | 2.1 (1.5-3) | 2.9e-05 | Risk |
| TCGA | SRSF2 | 0.7 | 2 (1.5-2.8) | 3.1e-05 | Risk |
| TCGA | RBM22 | 0.92 | 2.5 (1.6-3.9) | 3.3e-05 | Risk |
| TCGA | SNRNP40 | 0.66 | 1.9 (1.4-2.6) | 3.6e-05 | Risk |
| TCGA | PHF5A | 0.67 | 2 (1.4-2.7) | 4e-05 | Risk |
| TCGA | SRSF10 | 0.8 | 2.2 (1.5-3.3) | 0.00011 | Risk |
| TCGA | ALYREF | 0.38 | 1.5 (1.2-1.8) | 0.00015 | Risk |
| TCGA | SF3B6 | 0.65 | 1.9 (1.4-2.7) | 0.00015 | Risk |
| TCGA | EIF4A3 | 0.57 | 1.8 (1.3-2.4) | 0.00016 | Risk |
| TCGA | PPIH | 0.57 | 1.8 (1.3-2.4) | 0.00019 | Risk |
| TCGA | PPIL1 | 0.5 | 1.6 (1.3-2.1) | 0.00019 | Risk |
| TCGA | SNRPD1 | 0.43 | 1.5 (1.2-1.9) | 0.00024 | Risk |
| TCGA | HSPA8 | 0.45 | 1.6 (1.2-2) | 0.00026 | Risk |
| TCGA | SNRPB2 | 0.58 | 1.8 (1.3-2.5) | 0.00027 | Risk |
| TCGA | PRPF3 | 0.46 | 1.6 (1.2-2) | 3e-04 | Risk |
| TCGA | THOC2 | 0.57 | 1.8 (1.3-2.4) | 0.00032 | Risk |
| TCGA | TXNL4A | 0.48 | 1.6 (1.2-2.1) | 0.00033 | Risk |
| TCGA | SNRPA | 0.41 | 1.5 (1.2-1.9) | 0.00037 | Risk |
| TCGA | SNRPB | 0.35 | 1.4 (1.2-1.7) | 0.00052 | Risk |
| TCGA | U2AF2 | 0.57 | 1.8 (1.3-2.4) | 0.00056 | Risk |
| TCGA | HNRNPA3 | 0.54 | 1.7 (1.3-2.3) | 0.00059 | Risk |
| TCGA | SF3B2 | 0.69 | 2 (1.3-3) | 0.00076 | Risk |
| TCGA | SNW1 | 0.66 | 1.9 (1.3-2.8) | 0.00082 | Risk |
| TCGA | NCBP1 | 0.65 | 1.9 (1.3-2.8) | 0.00085 | Risk |
| TCGA | HNRNPU | 0.57 | 1.8 (1.3-2.5) | 0.0011 | Risk |
| TCGA | PRPF40B | 1.1 | 3.1 (1.6-6.2) | 0.0011 | Risk |
| TCGA | SMNDC1 | 0.66 | 1.9 (1.3-2.9) | 0.0011 | Risk |
| TCGA | SRSF7 | 0.61 | 1.8 (1.3-2.7) | 0.0011 | Risk |
| TCGA | DHX8 | 0.59 | 1.8 (1.3-2.6) | 0.0014 | Risk |
| TCGA | DDX23 | 0.57 | 1.8 (1.2-2.5) | 0.0016 | Risk |
| TCGA | HNRNPK | 0.65 | 1.9 (1.3-2.9) | 0.0016 | Risk |
| TCGA | SNRPA1 | 0.45 | 1.6 (1.2-2.1) | 0.0017 | Risk |
| TCGA | TCERG1 | 0.52 | 1.7 (1.2-2.3) | 0.0017 | Risk |
| TCGA | THOC1 | 0.6 | 1.8 (1.3-2.7) | 0.0017 | Risk |
| TCGA | SRSF3 | 0.56 | 1.8 (1.2-2.5) | 0.0018 | Risk |
| TCGA | PPIE | 0.6 | 1.8 (1.2-2.7) | 0.0021 | Risk |
| TCGA | CRNKL1 | 0.59 | 1.8 (1.2-2.6) | 0.0023 | Risk |
| TCGA | SNRPC | 0.38 | 1.5 (1.1-1.9) | 0.0023 | Risk |
| TCGA | SNU13 | 0.46 | 1.6 (1.2-2.1) | 0.0023 | Risk |
| TCGA | ISY1 | 0.46 | 1.6 (1.2-2.1) | 0.0024 | Risk |
| TCGA | PRPF40A | 0.51 | 1.7 (1.2-2.3) | 0.0024 | Risk |
| TCGA | SNRPG | 0.47 | 1.6 (1.2-2.2) | 0.0025 | Risk |
| TCGA | HNRNPC | 0.59 | 1.8 (1.2-2.7) | 0.0028 | Risk |
| TCGA | U2SURP | 0.45 | 1.6 (1.2-2.1) | 0.0028 | Risk |
| TCGA | BCAS2 | 0.55 | 1.7 (1.2-2.5) | 0.0033 | Risk |
| TCGA | SF3A2 | 0.35 | 1.4 (1.1-1.8) | 0.0035 | Risk |
| TCGA | SRSF9 | 0.44 | 1.5 (1.2-2.1) | 0.0038 | Risk |
| TCGA | BUD31 | 0.5 | 1.7 (1.2-2.3) | 0.0043 | Risk |
| TCGA | SART1 | 0.52 | 1.7 (1.2-2.4) | 0.0043 | Risk |
| TCGA | TRA2B | 0.62 | 1.9 (1.2-2.8) | 0.0044 | Risk |
| TCGA | LSM5 | 0.41 | 1.5 (1.1-2) | 0.0046 | Risk |
| TCGA | RBMX | 0.46 | 1.6 (1.2-2.2) | 0.0046 | Risk |
| TCGA | PRPF18 | 0.68 | 2 (1.2-3.1) | 0.0047 | Risk |
| TCGA | DHX15 | 0.42 | 1.5 (1.1-2.1) | 0.0055 | Risk |
| TCGA | SNRNP200 | 0.39 | 1.5 (1.1-1.9) | 0.0058 | Risk |
| TCGA | HSPA6 | 0.2 | 1.2 (1.1-1.4) | 0.0064 | Risk |
| TCGA | SLU7 | 0.6 | 1.8 (1.2-2.8) | 0.0064 | Risk |
| TCGA | ACIN1 | 0.47 | 1.6 (1.1-2.2) | 0.0066 | Risk |
| TCGA | DDX46 | 0.5 | 1.6 (1.1-2.4) | 0.0066 | Risk |
| TCGA | PRPF6 | 0.4 | 1.5 (1.1-2) | 0.0083 | Risk |
| TCGA | MAGOH | 0.45 | 1.6 (1.1-2.2) | 0.0084 | Risk |
| TCGA | SF3B3 | 0.44 | 1.5 (1.1-2.2) | 0.0096 | Risk |
| TCGA | SRSF1 | 0.51 | 1.7 (1.1-2.5) | 0.0097 | Risk |
| TCGA | PRPF38B | 0.37 | 1.5 (1.1-1.9) | 0.01 | Risk |
| TCGA | LSM2 | 0.28 | 1.3 (1.1-1.7) | 0.011 | Risk |
| TCGA | LSM4 | 0.27 | 1.3 (1.1-1.6) | 0.012 | Risk |
| TCGA | PRPF4 | 0.45 | 1.6 (1.1-2.2) | 0.012 | Risk |
| TCGA | SF3B5 | 0.27 | 1.3 (1-1.6) | 0.017 | Risk |
| TCGA | DDX42 | 0.44 | 1.6 (1.1-2.2) | 0.019 | Risk |
| TCGA | SF3A1 | 0.37 | 1.5 (1.1-2) | 0.019 | Risk |
| TCGA | TRA2A | 0.41 | 1.5 (1.1-2.1) | 0.019 | Risk |
| TCGA | SNRPF | 0.3 | 1.3 (1-1.7) | 0.02 | Risk |
| TCGA | SF3B1 | 0.34 | 1.4 (1-1.9) | 0.023 | Risk |
| TCGA | SNRPE | 0.25 | 1.3 (1-1.6) | 0.025 | Risk |
| TCGA | PUF60 | 0.26 | 1.3 (1-1.6) | 0.027 | Risk |
| TCGA | SRSF6 | 0.43 | 1.5 (1-2.3) | 0.027 | Risk |
| TCGA | DHX38 | 0.34 | 1.4 (1-1.9) | 0.028 | Risk |
| TCGA | PQBP1 | 0.28 | 1.3 (1-1.7) | 0.031 | Risk |
| TCGA | RBM8A | 0.35 | 1.4 (1-2) | 0.037 | Risk |
| TCGA | SRSF4 | 0.39 | 1.5 (1-2.1) | 0.037 | Risk |
| TCGA | RBM25 | 0.36 | 1.4 (1-2) | 0.038 | Risk |
| TCGA | SNRNP27 | 0.4 | 1.5 (1-2.2) | 0.044 | Risk |
| TCGA | HNRNPA1 | 0.27 | 1.3 (1-1.7) | 0.046 | Risk |
| TCGA | WBP11 | 0.33 | 1.4 (1-1.9) | 0.047 | Risk |
| ICGC | SNRPD1 | 0.57 | 1.8 (1.3-2.5) | 0.0011 | Risk |
| ICGC | LSM2 | 0.57 | 1.8 (1.2-2.6) | 0.003 | Risk |
| ICGC | SNRPB | 0.53 | 1.7 (1.2-2.4) | 0.0031 | Risk |
| ICGC | SNRPF | 0.62 | 1.9 (1.2-2.8) | 0.0031 | Risk |
| ICGC | TXNL4A | 0.59 | 1.8 (1.2-2.7) | 0.0033 | Risk |
| ICGC | SNRPG | 0.6 | 1.8 (1.2-2.8) | 0.0051 | Risk |
| ICGC | LSM5 | 0.58 | 1.8 (1.1-2.8) | 0.011 | Risk |
| ICGC | SNRPA1 | 0.54 | 1.7 (1.1-2.6) | 0.011 | Risk |
| ICGC | LSM4 | 0.4 | 1.5 (1.1-2) | 0.013 | Risk |
| ICGC | PPIL1 | 0.38 | 1.5 (1.1-2) | 0.02 | Risk |
| ICGC | SRSF9 | 0.43 | 1.5 (1.1-2.2) | 0.025 | Risk |
| ICGC | PRPF19 | 0.45 | 1.6 (1-2.4) | 0.034 | Risk |
| ICGC | SNRPB2 | 0.47 | 1.6 (1-2.5) | 0.038 | Risk |
| ICGC | SF3A3 | 0.44 | 1.5 (1-2.3) | 0.04 | Risk |
| ICGC | SNRPD2 | 0.35 | 1.4 (1-2) | 0.043 | Risk |
| ICGC | EIF4A3 | 0.38 | 1.5 (1-2.1) | 0.044 | Risk |

**Table S5. Relationship between SNRPB expression and tumor characteristics in patients with HCC analyzed by Spearman rank correlation analysis**

| Tumor characteristics | index |  |
| --- | --- | --- |
| T Infiltrate | Pearson correlation | 0.238 |
|  | Significance (two tailed) | 0.037 |
|  | n | 77 |
| Stage | Pearson correlation | 0.266 |
|  | Significance (two tailed) | 0.019 |
|  | n | 77 |

**Table S6 The risk genes identified by univariate Cox regression analyses for cell cycle genes in TCGA and ICGC cohorts.**

| **Database** | **Gene** | **Coef** | **HR (95% CI)** | **P.value** | **Type** |
| --- | --- | --- | --- | --- | --- |
| TCGA | CDC20 | 0.33 | 1.4 (1.2-1.6) | 1.3e-07 | risk |
| TCGA | PLK1 | 0.48 | 1.6 (1.3-1.9) | 3.3e-07 | risk |
| TCGA | CCNB1 | 0.37 | 1.4 (1.2-1.7) | 1.7e-06 | risk |
| TCGA | ORC6 | 0.6 | 1.8 (1.4-2.3) | 4.2e-06 | risk |
| TCGA | MCM6 | 0.43 | 1.5 (1.3-1.8) | 4.3e-06 | risk |
| TCGA | ORC1 | 0.5 | 1.7 (1.3-2.1) | 5.3e-06 | risk |
| TCGA | CDK1 | 0.36 | 1.4 (1.2-1.7) | 9e-06 | risk |
| TCGA | CDC45 | 0.43 | 1.5 (1.3-1.9) | 1.4e-05 | risk |
| TCGA | BUB1B | 0.48 | 1.6 (1.3-2) | 2.1e-05 | risk |
| TCGA | CDC25A | 0.5 | 1.7 (1.3-2.1) | 2.3e-05 | risk |
| TCGA | CDC7 | 0.51 | 1.7 (1.3-2.1) | 3e-05 | risk |
| TCGA | CDC6 | 0.36 | 1.4 (1.2-1.7) | 4.1e-05 | risk |
| TCGA | CDC25B | 0.36 | 1.4 (1.2-1.7) | 4.3e-05 | risk |
| TCGA | CCNA2 | 0.28 | 1.3 (1.2-1.5) | 4.7e-05 | risk |
| TCGA | CDK4 | 0.41 | 1.5 (1.2-1.8) | 5.6e-05 | risk |
| TCGA | MCM2 | 0.32 | 1.4 (1.2-1.6) | 5.6e-05 | risk |
| TCGA | CDC25C | 0.42 | 1.5 (1.2-1.9) | 6.6e-05 | risk |
| TCGA | MCM4 | 0.36 | 1.4 (1.2-1.7) | 0.00013 | risk |
| TCGA | SFN | 0.15 | 1.2 (1.1-1.3) | 0.00018 | risk |
| TCGA | PRKDC | 0.43 | 1.5 (1.2-1.9) | 0.00022 | risk |
| TCGA | CDK2 | 0.46 | 1.6 (1.2-2) | 0.00028 | risk |
| TCGA | SKP2 | 0.5 | 1.7 (1.3-2.2) | 0.00034 | risk |
| TCGA | CCNB2 | 0.29 | 1.3 (1.1-1.6) | 0.00044 | risk |
| TCGA | CCNE2 | 0.63 | 1.9 (1.3-2.7) | 0.00047 | risk |
| TCGA | PCNA | 0.37 | 1.4 (1.2-1.8) | 0.00052 | risk |
| TCGA | MCM7 | 0.29 | 1.3 (1.1-1.6) | 0.00054 | risk |
| TCGA | PKMYT1 | 0.43 | 1.5 (1.2-2) | 0.00062 | risk |
| TCGA | MCM5 | 0.35 | 1.4 (1.2-1.7) | 0.00064 | risk |
| TCGA | MCM3 | 0.31 | 1.4 (1.1-1.6) | 0.00084 | risk |
| TCGA | TFDP1 | 0.34 | 1.4 (1.2-1.7) | 0.00086 | risk |
| TCGA | E2F1 | 0.22 | 1.2 (1.1-1.4) | 0.0012 | risk |
| TCGA | WEE1 | 0.33 | 1.4 (1.1-1.7) | 0.0057 | risk |
| TCGA | SMC1A | 0.3 | 1.4 (1.1-1.7) | 0.011 | risk |
| ICGC | CDC20 | 0.41 | 1.5 (1.2-1.8) | 3.5e-05 | risk |
| ICGC | CCNB1 | 0.53 | 1.7 (1.3-2.2) | 3.8e-05 | risk |
| ICGC | CDC6 | 0.42 | 1.5 (1.2-1.9) | 5.1e-05 | risk |
| ICGC | E2F1 | 0.43 | 1.5 (1.2-1.9) | 9.2e-05 | risk |
| ICGC | CDC25A | 0.4 | 1.5 (1.2-1.8) | 0.00012 | risk |
| ICGC | CDC25C | 0.46 | 1.6 (1.3-2) | 0.00013 | risk |
| ICGC | CDK1 | 0.49 | 1.6 (1.3-2.1) | 0.00014 | risk |
| ICGC | MCM2 | 0.41 | 1.5 (1.2-1.9) | 0.00014 | risk |
| ICGC | PLK1 | 0.41 | 1.5 (1.2-1.9) | 0.00015 | risk |
| ICGC | CDC45 | 0.42 | 1.5 (1.2-1.9) | 0.00016 | risk |
| ICGC | BUB1B | 0.4 | 1.5 (1.2-1.9) | 0.00021 | risk |
| ICGC | CCNA2 | 0.5 | 1.6 (1.3-2.1) | 0.00024 | risk |
| ICGC | CDK4 | 0.63 | 1.9 (1.3-2.6) | 0.00024 | risk |
| ICGC | CCNB2 | 0.37 | 1.5 (1.2-1.8) | 0.00078 | risk |
| ICGC | MCM6 | 0.45 | 1.6 (1.2-2.1) | 0.0011 | risk |
| ICGC | ORC6 | 0.35 | 1.4 (1.1-1.8) | 0.0016 | risk |
| ICGC | CCNE2 | 0.37 | 1.4 (1.1-1.8) | 0.0019 | risk |
| ICGC | MCM5 | 0.45 | 1.6 (1.2-2.1) | 0.0021 | risk |
| ICGC | MCM4 | 0.37 | 1.4 (1.1-1.8) | 0.0026 | risk |
| ICGC | MCM7 | 0.5 | 1.6 (1.2-2.3) | 0.0032 | risk |
| ICGC | PKMYT1 | 0.33 | 1.4 (1.1-1.7) | 0.0034 | risk |
| ICGC | ORC1 | 0.33 | 1.4 (1.1-1.8) | 0.0048 | risk |
| ICGC | MCM3 | 0.39 | 1.5 (1.1-1.9) | 0.0052 | risk |
| ICGC | SFN | 0.15 | 1.2 (1-1.3) | 0.0071 | risk |
| ICGC | CDK2 | 0.43 | 1.5 (1.1-2.2) | 0.015 | risk |
| ICGC | PCNA | 0.4 | 1.5 (1.1-2.1) | 0.017 | risk |
| ICGC | SMC1A | 0.41 | 1.5 (1.1-2.1) | 0.018 | risk |
| ICGC | TFDP1 | 0.31 | 1.4 (1-1.8) | 0.027 | risk |
| ICGC | CDC25B | 0.31 | 1.4 (1-1.8) | 0.044 | risk |
| ICGC | CDC7 | 0.25 | 1.3 (1-1.6) | 0.047 | risk |

**Table S7 The transcription factors in downregulated genes.**

| **Species** | **Symbol** | **Ensembl** | **Family** | **Protein** | **Entrez ID** |
| --- | --- | --- | --- | --- | --- |
| Homo_sapiens | MYBL2 | ENSG00000101057 | MYB | ENSP00000380072;ENSP00000217026; | 4605 |
| Homo_sapiens | TADA2A | ENSG00000276234 | MYB | ENSP00000484884;ENSP00000477709;ENSP00000480446;ENSP00000478363;ENSP00000481890;ENSP00000478162;ENSP00000481091; | 6871 |
| Homo_sapiens | NFYB | ENSG00000120837 | NF-YB | ENSP00000448250;ENSP00000447486;ENSP00000240055; | 4801 |
| Homo_sapiens | TFDP1 | ENSG00000198176 | E2F | ENSP00000364519;ENSP00000386145;ENSP00000401389; | 7027 |
| Homo_sapiens | ZNF512 | ENSG00000243943 | zf-C2H2 | ENSP00000347648;ENSP00000451572;ENSP00000407038;ENSP00000369040;ENSP00000395660; | 84450 |
| Homo_sapiens | FOXE1 | ENSG00000178919 | Fork_head | ENSP00000364265; | 2304 |
| Homo_sapiens | NFYC | ENSG00000066136 | NF-YC | ENSP00000436710;ENSP00000361738;ENSP00000361734;ENSP00000409219;ENSP00000396620;ENSP00000361737;ENSP00000416403;ENSP00000408867;ENSP00000408315;ENSP00000312617;ENSP00000433820;ENSP00000397647;ENSP00000433413;ENSP00000414299;ENSP00000434404;ENSP00000361736;ENSP00000404427;ENSP00000361754; | 4802 |
| Homo_sapiens | GTF2I | ENSG00000263001 | GTF2I | ENSP00000477837;ENSP00000460070;ENSP00000484526;ENSP00000482476;ENSP00000404240; | 2969 |
| Homo_sapiens | ZFP36L2 | ENSG00000152518 | zf-CCCH | ENSP00000282388; | 678 |
| Homo_sapiens | ID3 | ENSG00000117318 | bHLH | ENSP00000363689; | 3399 |
| Homo_sapiens | ZNF594 | ENSG00000180626 | zf-C2H2 | ENSP00000382513;ENSP00000461032; | 84622 |
| Homo_sapiens | SSRP1 | ENSG00000149136 | HMG | ENSP00000489564;ENSP00000278412; | 6749 |
| Homo_sapiens | NR2F2 | ENSG00000185551 | RXR-like | ENSP00000377721;ENSP00000377726;ENSP00000457112;ENSP00000389853;ENSP00000401674; | 7026 |
| Homo_sapiens | HMGB3 | ENSG00000029993 | HMG | ENSP00000410354;ENSP00000359393;ENSP00000405601;ENSP00000417027;ENSP00000442758; | 3149 |
| Homo_sapiens | E2F1 | ENSG00000101412 | E2F | ENSP00000345571; | 1869 |
| Homo_sapiens | GBX2 | ENSG00000168505 | Homeobox | ENSP00000302251; | 2637 |
| Homo_sapiens | TP53 | ENSG00000141510 | P53 | ENSP00000425104;ENSP00000482903;ENSP00000481401;ENSP00000478219;ENSP00000481638;ENSP00000477531;ENSP00000481179;ENSP00000426252;ENSP00000482258;ENSP00000352610;ENSP00000473895;ENSP00000391127;ENSP00000424104;ENSP00000398846;ENSP00000484375;ENSP00000269305;ENSP00000410739;ENSP00000480868;ENSP00000478499;ENSP00000482222;ENSP00000423862;ENSP00000482537;ENSP00000488924;ENSP00000484409;ENSP00000391478; | 7157 |
| Homo_sapiens | MYBL1 | ENSG00000185697 | MYB | ENSP00000429633;ENSP00000428011;ENSP00000428265; | 4603 |
| Homo_sapiens | E2F8 | ENSG00000129173 | E2F | ENSP00000481103;ENSP00000434199;ENSP00000250024; | 79733 |
| Homo_sapiens | FOXM1 | ENSG00000111206 | Fork_head | ENSP00000342307;ENSP00000354492;ENSP00000442309;ENSP00000352901;ENSP00000486536; | 2305 |
| Homo_sapiens | WDHD1 | ENSG00000198554 | HMG | ENSP00000399349;ENSP00000353793; | 11169 |
| Homo_sapiens | HOXC6 | ENSG00000197757 | Homeobox | ENSP00000243108;ENSP00000377864; | 3223 |
| Homo_sapiens | TEAD4 | ENSG00000197905 | TEA | ENSP00000444528;ENSP00000352926;ENSP00000438453;ENSP00000351184;ENSP00000411475; | 7004 |
| Homo_sapiens | CENPA | ENSG00000115163 | Others | - | 1058 |
| Homo_sapiens | TCF19 | ENSG00000137310 | Others | - | 6941 |

**Table S8. Expression patterns of CCNB1 in HCC tissues and normal tissues revealed in immunohistochemistry analysis**

| CCNB1 expression | Tumor tissue | | Normal tissue | |
| --- | --- | --- | --- | --- |
|  | Cases | Percentage | Cases | Percentage |
| Low | 37 | 51.4% | 84 | 100.0% |
| High | 35 | 48.6% | 0 | 0.0% |

*P* < 0.001

**Table S9. Relationship between CCNB1 expression and tumor characteristics in patients with HCC**

| Features | No. of patients | CCNB1 expression | | *P* value |
| --- | --- | --- | --- | --- |
|  |  | low | high |  |
| All patients | 72 | 37 | 35 |  |
| Age (years) |  |  |  | 0.005 |
| <58 | 35 | 12 | 23 |  |
| ≥58 | 37 | 25 | 12 |  |
| Gender |  |  |  | 0.464 |
| Male | 60 | 32 | 28 |  |
| Female | 12 | 5 | 7 |  |
| Grade |  |  |  | 0.035 |
| II | 36 | 23 | 13 |  |
| III | 36 | 14 | 22 |  |
| Tumor size |  |  |  | 0.474 |
| <6 cm | 34 | 19 | 15 |  |
| ≥6 cm | 38 | 18 | 20 |  |
| Stage |  |  |  | 0.145 |
| 1 | 25 | 16 | 9 |  |
| 2 | 31 | 14 | 17 |  |
| 3 | 15 | 7 | 8 |  |
| 4 | 1 | 0 | 1 |  |
| T Infiltrate |  |  |  | 0.224 |
| T1 | 25 | 16 | 9 |  |
| T2 | 32 | 14 | 18 |  |
| T3 | 6 | 2 | 4 |  |
| T4 | 9 | 5 | 4 |  |
|  |  |  |  |  |

**Table S10. Relationship between CCNB1 expression and tumor characteristics in patients with HCC analyzed by Spearman rank correlation analysis**

| Tumor characteristics | index |  |
| --- | --- | --- |
| Age (years) | Pearson correlation | -0.333 |
|  | Significance (two tailed) | 0.004 |
|  | n | 72 |
| Grade | Pearson correlation | 0.085 |
|  | Significance (two tailed) | 0.478 |
|  | n | 72 |
