# Supplementary material for: SNRPB/CCNB1 axis promotes hepatocellular carcinoma progression and cisplatin resistance through enhancing lipid metabolism reprogramming
Source: J Exp Clin Cancer Res. 2025 Jul 18;44:211. doi: 10.1186/s13046-025-03463-y (PMC12273286; doi:10.1186/s13046-025-03463-y)

**Figure S1. (A) The featureplot showing expression of oncogenes in global cell types. (B) The box plot showing the differences in expression levels between tumor and normal groups in TCGA and ICGC cohorts.**


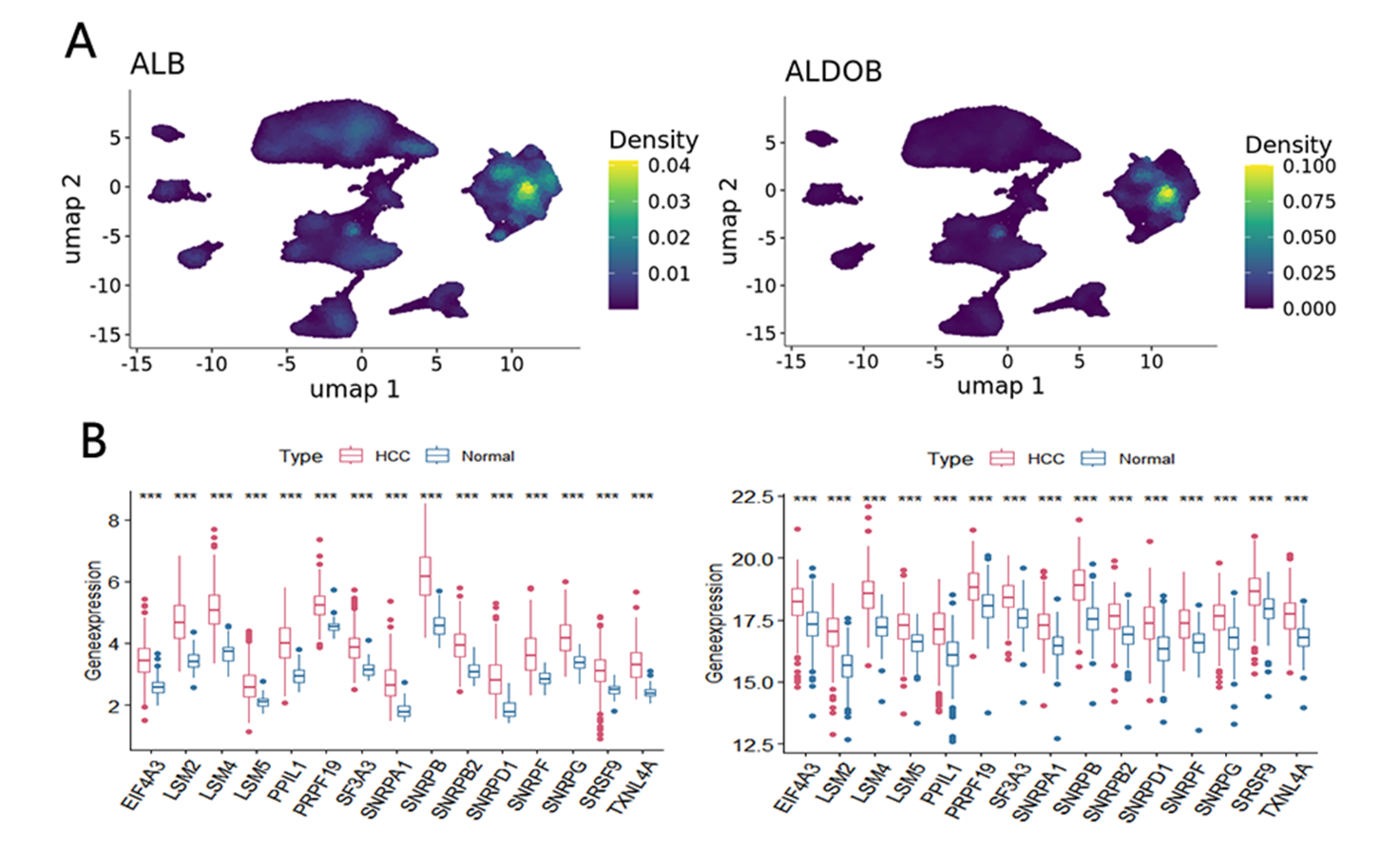


**Figure S2. (A) The heatmap illustrating the CNV profiles for each epithelial cell cluster in the tumor, with red and blue hues indicating high and low CNV levels, respectively. Epithelial cells derived from normal tissue serves as the reference. (B) The box plot showing the CNV scores of different epithelial cell cluster in the tumor. (C) The spatial distribution of C3 cluster in HCC determined by spatial transcriptome data (HRA000437)**


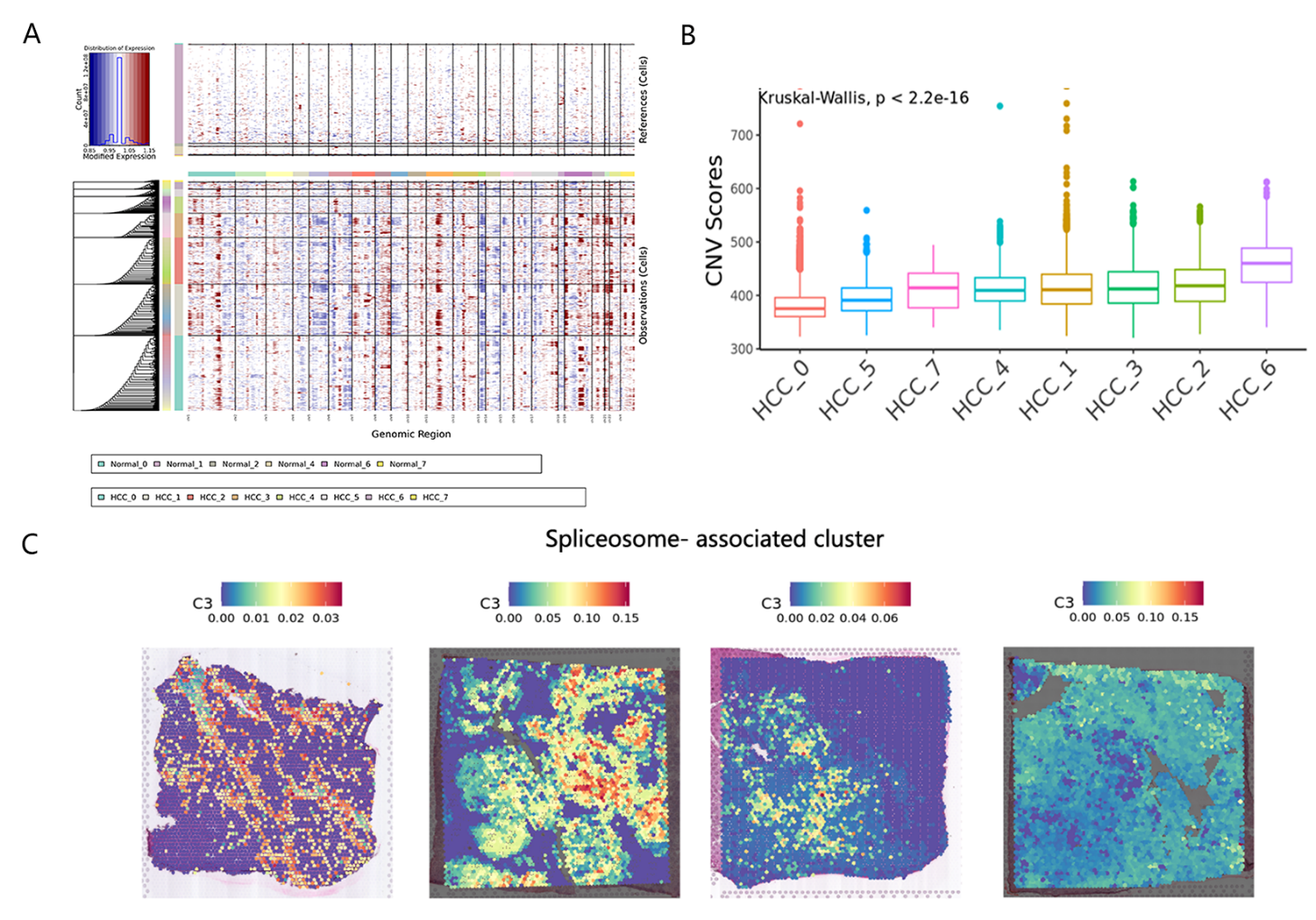


**Figure S3. (A) qPCR analysis revealed significantly higher SNRPB mRNA expression in HCC tissues compared to adjacent normal tissues. (B) The box plot showing elevated SNRPB expression in HCC compared to normal liver tissues in multiple datasets. (C) Survival analysis showed that elevated SNRPB expression were associated with poor prognosis in HCC patients in FUSCC cohort.**


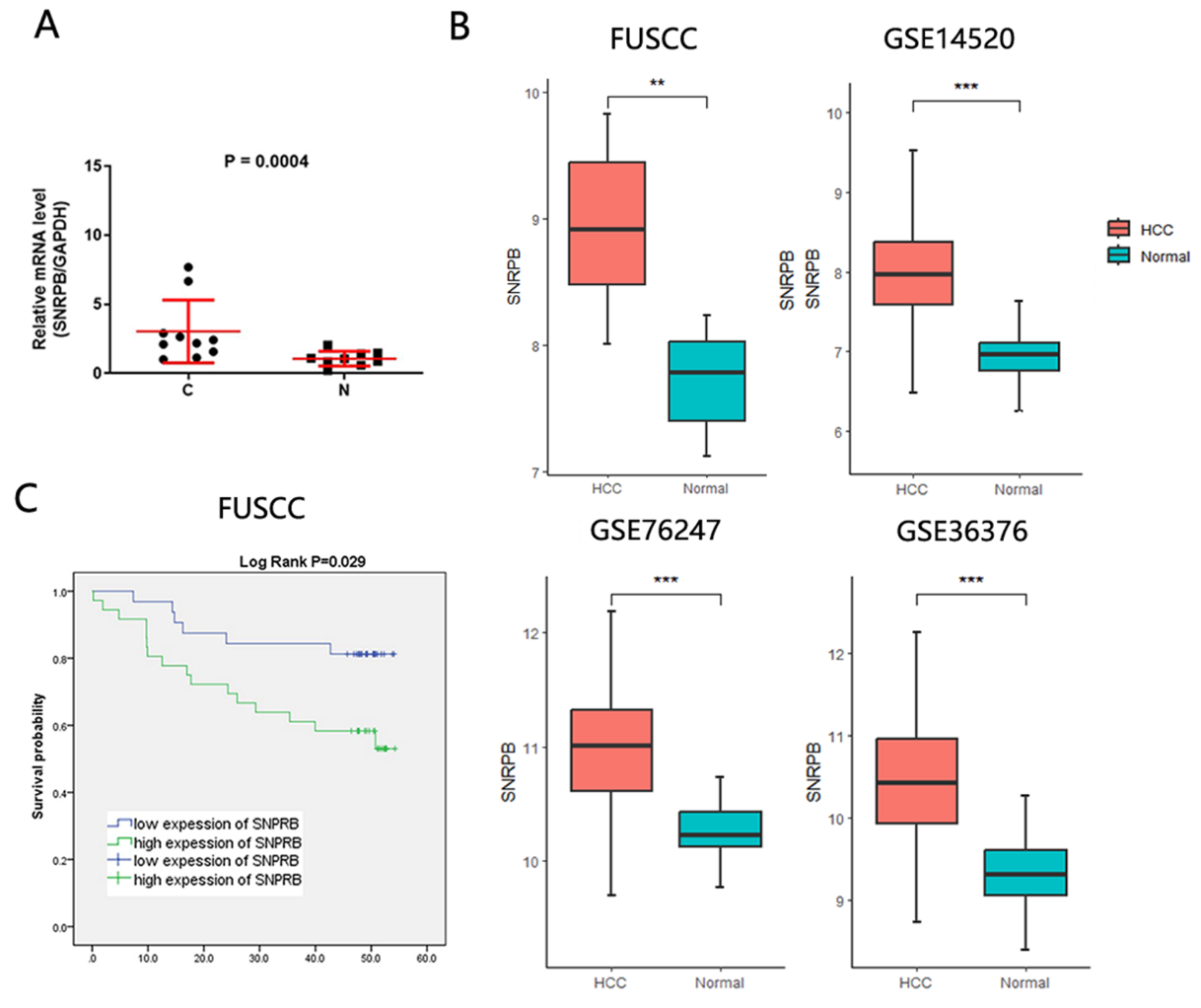


**Figure S4. The knockdown efficiencies of 3 shRNAs to knock down the expression of SNRPB were evaluated in SK-HEP-1 cells on mRNA and protein levels.** Data were drawn as mean ± SD (n ≥ 3). ** P < 0.01, *** P < 0.001


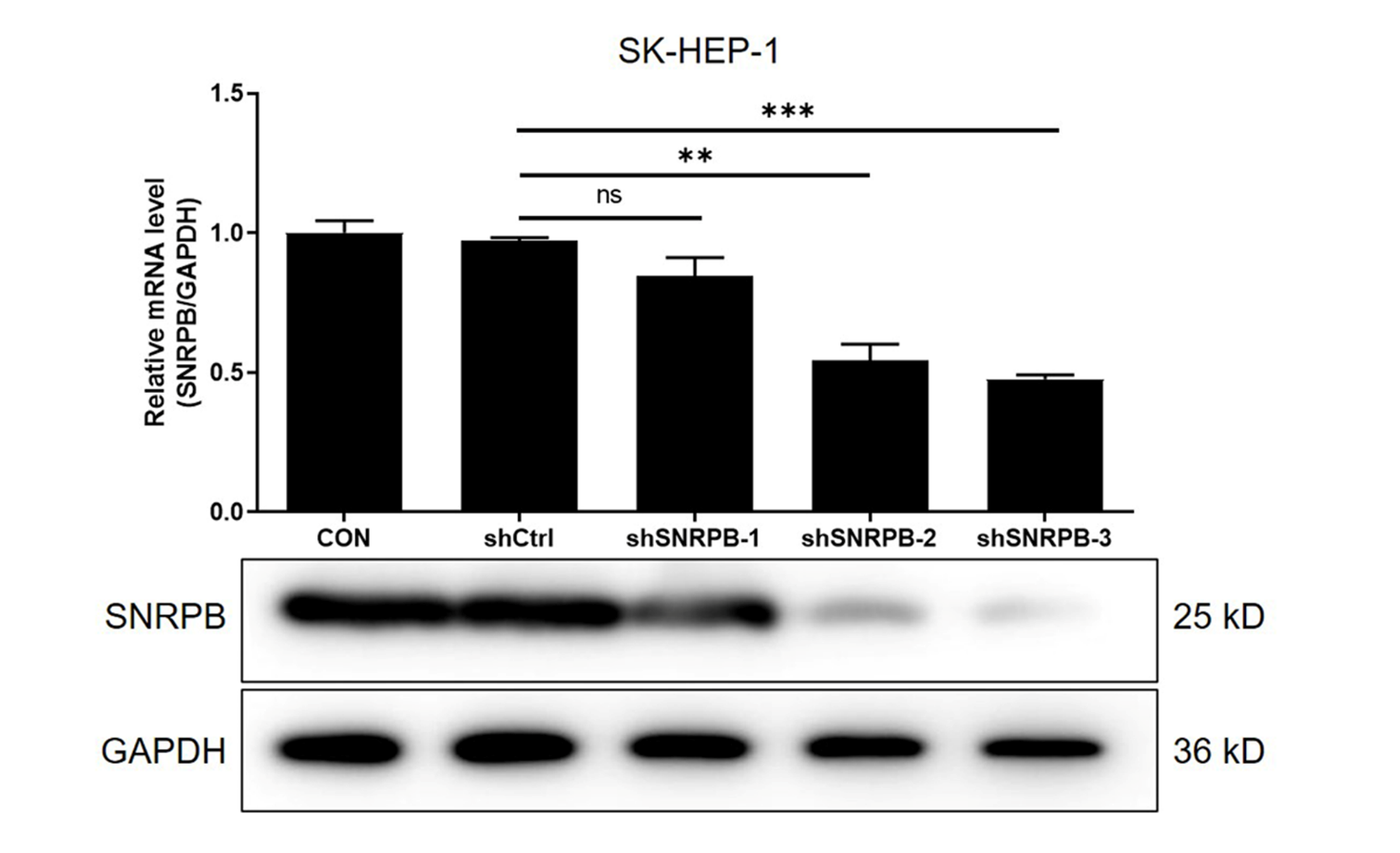


**Figure S5. (A) Wound-healing assay demonstrates that silencing SNRPB significantly reduces the migratory capacity of HCC cells. (B) The expression of EMT-related proteins including E-cadherin, N-cadherin, Vimentin and Snail was detected in HCCLM3 and SK-HEP-1 cells with or without SNRPB knockdown.** Data were drawn as mean ± SD (n ≥ 3). * P < 0.05, *** P < 0.001


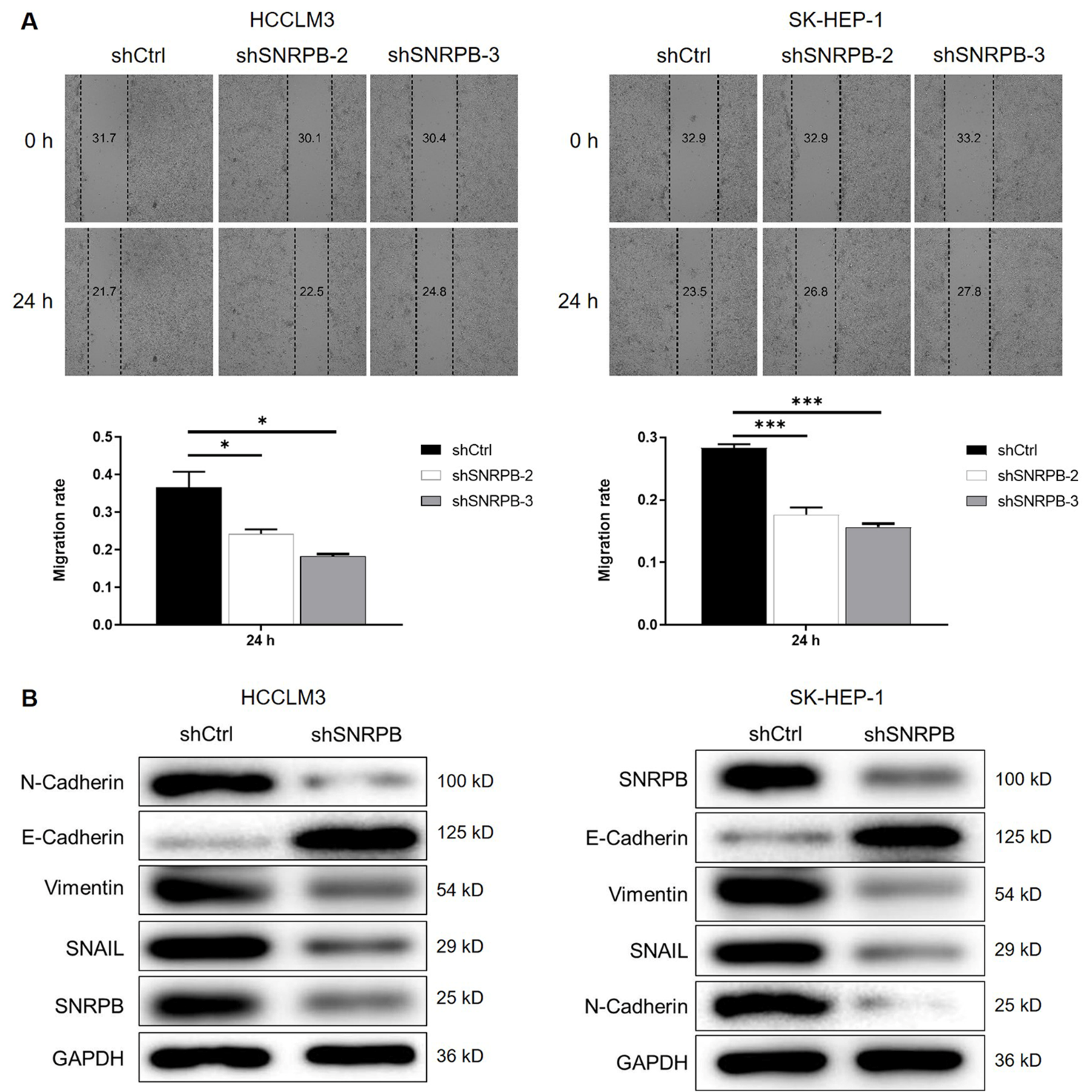


**Figure S6. (A) The Venn plot showed the number of transcription factors in the downregulated genes. (B) The Protein-Protein Interaction Networks between transcription factors and SNRPB. (C) The protein levels of SNRPB and CCNB1 in HCCLM3 and SK-HEP-1 cells with or without SNRPB knockdown were detected by WB. (D) Endogenous CCNB1 expression was compared in normal liver cells (HL-7702) and HCC cell lines, with significantly higher levels observed in HCC cells.** Data were drawn as mean ± SD (n ≥ 3). * P < 0.05, *** P < 0.001. **(E) The box plot showing elevated CCNB1 expression in HCC compared to normal liver tissues in FUSCC cohort. (F) Survival analysis showed that elevated CCNB1 expression were associated with poor prognosis in HCC patients in FUSCC cohort.**


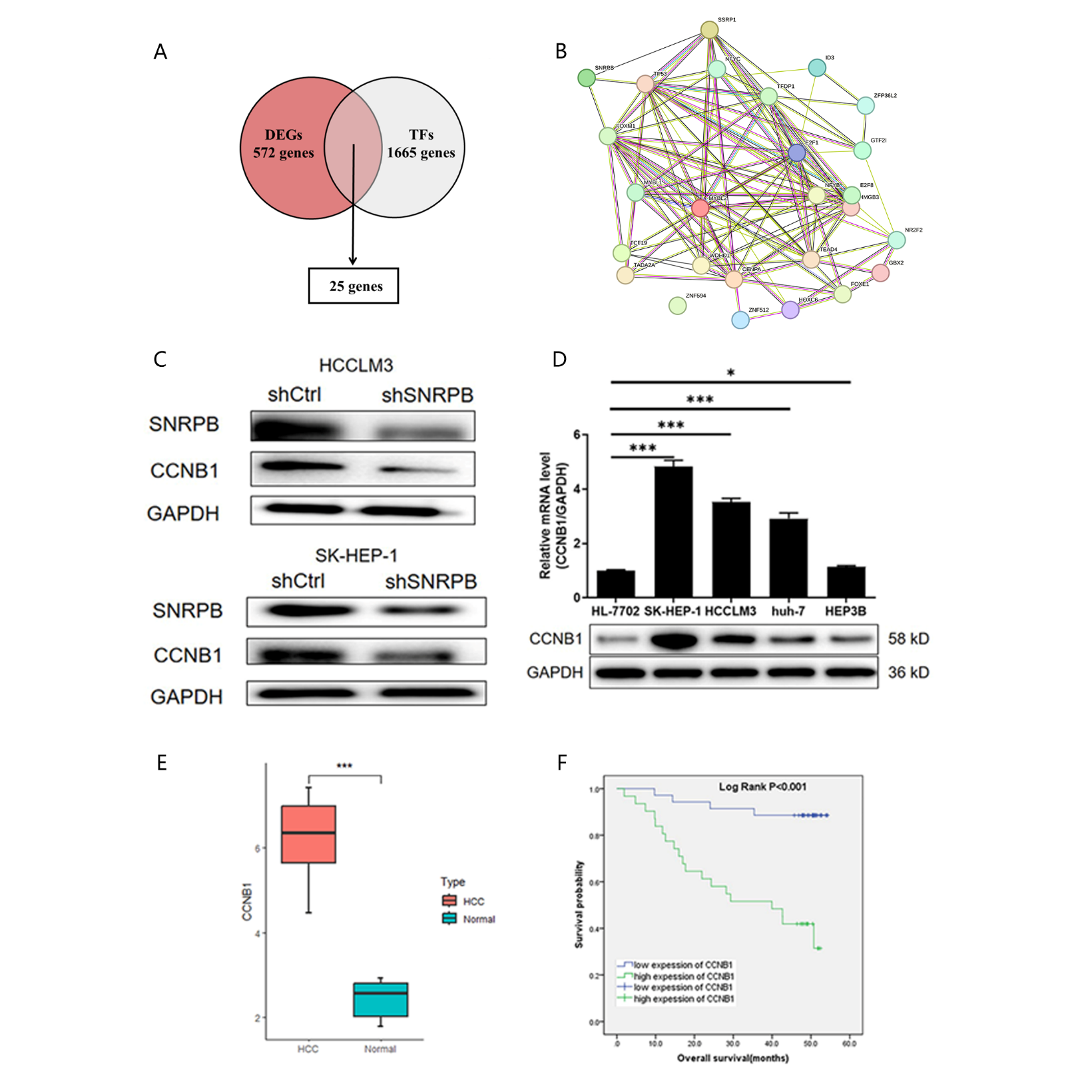


**Figure S7. (A) The triglyceride, total cholesterol, and free fatty acid levels were detected in HCCLM3 cells with indicated treatment. (B) The triglyceride, total cholesterol, and free fatty acid levels were detected in SK-HEP-1 cells with indicated treatment.** Data were drawn as mean ± SD (n ≥ 3). * P < 0.05, ** P < 0.01, *** P < 0.001


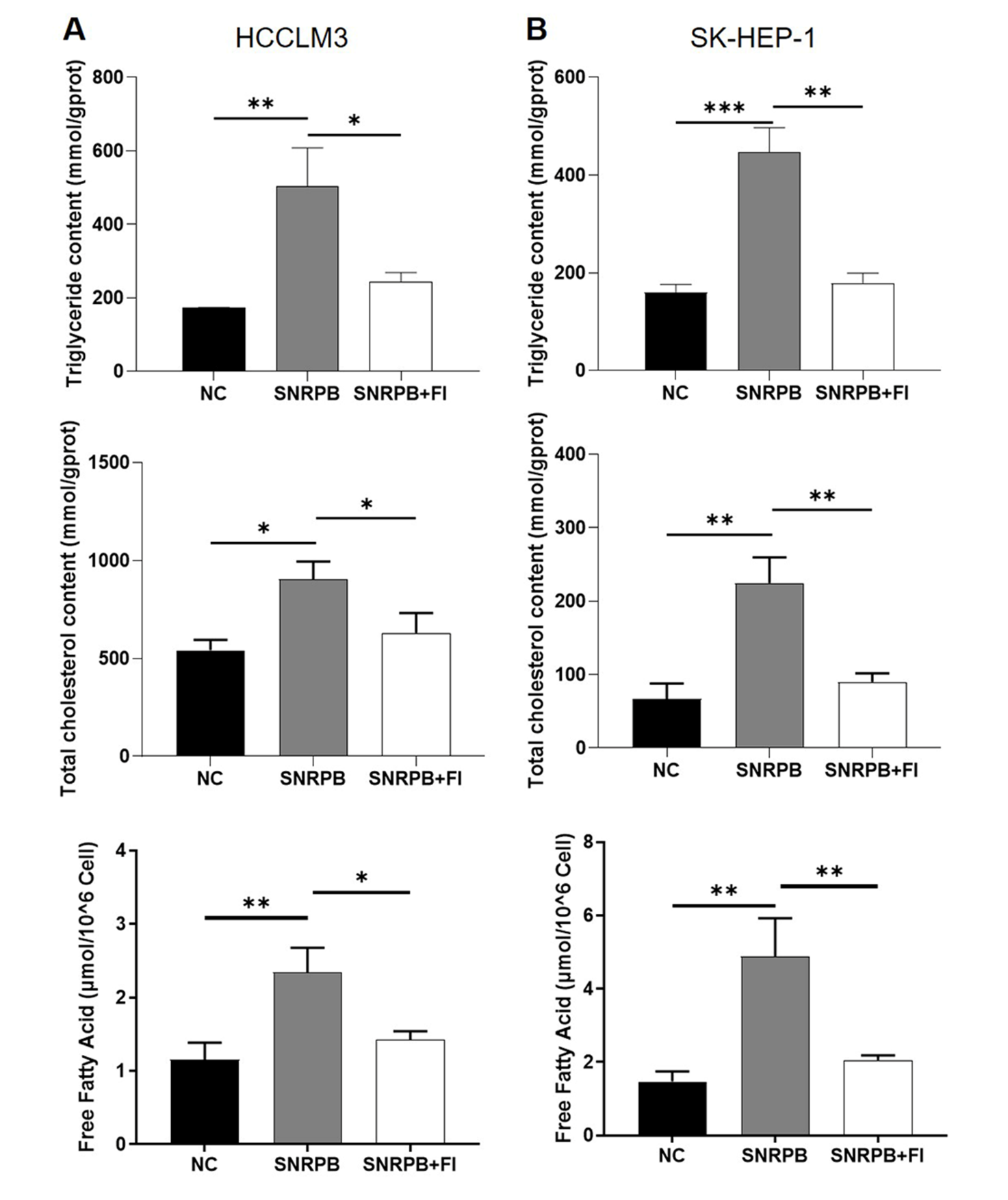

Supplement: Supplementary file 2 — Supplementary Material 2 [file 13046_2025_3463_MOESM2_ESM.docx]
